# Clinical translation and landscape of stimuli-responsive nanomedicines and microscale therapeutics

**DOI:** 10.1039/d6cs00165c

**Published:** 2026-05-29

**Authors:** Dmytro Kobzev, Olesia Kulyk, Roman A. Barmin, Anatoliy Tatarets, Roger M. Pallares, Fabian Kiessling, Twan Lammers, Quim Peña

**Affiliations:** a Institute for Experimental Molecular Imaging, Center for Biohybrid Medical Systems, RWTH Aachen University Clinic Aachen 52074 Germany dkobzev@ukaachen.de tlammers@ukaachen.de jpena@ukaachen.de; b Institute of Functional Materials Chemistry of State Scientific Institution “Institute for Single Crystals” of National Academy of Sciences of Ukraine Kharkiv 61072 Ukraine

## Abstract

Stimuli-responsive materials enable temporal and spatial control over drug delivery and action. Traditional triggerable therapeutics are largely based on small molecules, like prodrugs and photodynamic therapy agents. Advances in nanotechnology and micromaterials have greatly expanded the field, as evidenced by clinically translated hyperthermia-generating iron oxide nanoparticles, radiotherapy-enhancing hafnium oxide nanoparticles, and ultrasound-responsive microbubbles. We here analyze the (pre-)clinical landscape of trigger-responsive therapeutics between 2014 and 2024, encompassing over 90 000 publications and 1000 clinical trials. External stimuli include light, ultrasound, radiation, magnetic field and temperature. Key internal stimuli are pH, redox and enzymes. Our analysis shows that light is by far the most popular external stimulus (44% of papers; 361 trials). Among internal stimuli, which account for 46% of papers (558 trials), redox and enzyme activation are the most explored ones. In recent years, interest in radiation (114 trials), ultrasound (33 trials), temperature (14 trials), and magnetic actuation (3 trials) is increasing, typically involving nano- and microscale platforms. In the second part of our paper, we examine translational trajectories and identify key barriers that are limiting the clinical progress of stimuli-responsive therapeutics. Important issues to address to help promote clinical translation include: (1) inaccurate medical need identification; (2) overly complex material design; (3) limited tissue penetration; (4) limited device accessibility; (5) economic constraints; and (6) challenging clinical adoption. We conclude by providing practical and practicable solutions to address these key limitations, going from nano- and micro-formulation design to development, translation and implementation, together aiming to increase the clinical impact of stimuli-responsive therapeutics.

## Abstract 2

We present a large-scale scientometric (pre-)clinical landscape analysis of stimuli-responsive therapeutics, highlighting the impact of nano-/micro-scale material platforms, and mapping translational barriers and strategies to overcome them.

## Graphical abstract

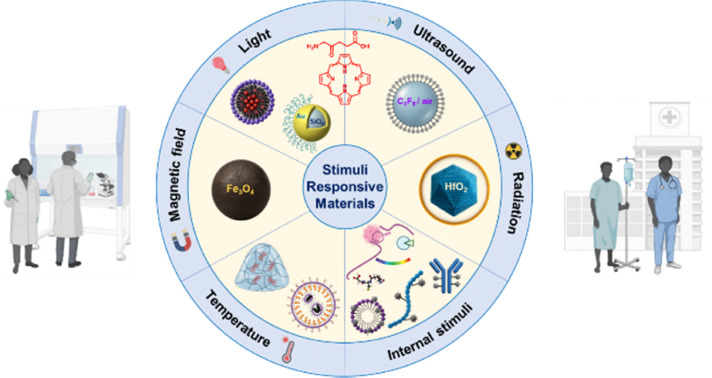

## 1. Introduction

Stimuli-responsive materials have garnered considerable interest for diagnostic and therapeutic purposes. With several products already in clinical use, such as the light-responsive drug Photofrin^1^ and the radiation-enhancing nanodrug Hensify,^2,3^ this type of materials are engineered to respond to external (light,^4–6^ temperature,^7,8^ radiation,^3,9^ magnetic field^10–12^ and ultrasound^13,14^) or internal stimuli (pH,^15,16^ redox^17^ and enzymes^16,18^), resulting in the induction of direct material-mediated therapeutic effects^16,17,19^ or triggering the release of active pharmaceutical ingredients (API). Many clinically relevant stimuli-responsive therapeutics are based on small molecules, such as fluorescent dyes and prodrugs.^5,9,14^ Yet, the increasing clinical relevance of nano- and micro-technologies in the past decades^20,21^ has also promoted the development and use of novel stimuli-responsive platforms for therapeutic purposes, like superparamagnetic iron oxide nanoparticles (SPION).^10–12^ Stimuli-responsive platforms allow to tailor pharmacokinetics and biodistribution profiles, enhance target-site accumulation, and increase stimuli-material responsiveness and performance control, thereby resulting in improved therapeutic outcomes.^22^

The conceptual roots of stimuli-responsive materials can be traced back more than a century to the early foundations of light-based medical therapies. Back in the 1880s, the physician Niels Finsen pioneered the use of phototherapy to treat *lupus vulgaris*, a skin condition caused by tuberculosis.^23^ His work, which earned the Nobel Prize in Medicine in 1903, laid the foundation for modern light-based therapies and led to the development of photodynamic therapy (PDT). In the 1960s, PDT was clinically established and has been used since then in the treatment of various cancers, including esophageal (*e.g.*, Photofrin) and prostate (*e.g.*, Foscan and TOOKAD),^1^ as well as other diseases such as actinic keratosis (*e.g.*, Metvix)^24^ and macular degeneration (*e.g.*, Visudyne).^25^ In fact, PDT global market share was valued at USD 4.6 billion in 2024, and it is predicted to double in the next 10 years,^26,27^ highlighting its continuously increasing interest. Other recent light-based treatments, such as photothermal therapy (PTT)^28^ and photoimmunotherapy (PIT),^29^ are currently under clinical evaluation (*e.g.*, gold nanoparticle-based product AuroShell^30^ and phthalocyanine-antibody conjugate ASP-1929).^29^

**Fig. 1:**
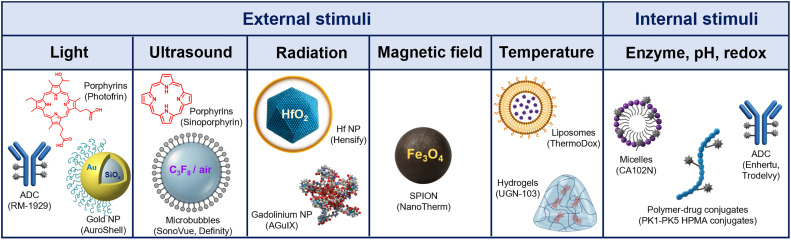
Representative examples of clinically relevant stimuli-responsive materials and products. Over the years, many different stimuli-responsive materials have been designed and evaluated for the treatment of several diseases, including cancer, inflammation, and infectious diseases. These mostly include small-molecule fluorescent dyes and prodrugs, which can also be delivered in nanoscale carriers based on lipids, polymers or antibodies, but also nano- and microscale platforms intrinsically responsive to external stimuli (*e.g.*, magnetic- or radiation-responsive metal-based nanoparticles and ultrasound-responsive microbubbles). *Abbreviations*: NP – nanoparticles, SPION – superparamagnetic iron oxide nanoparticles, Hf – hafnium, ADC – antibody–drug conjugates, HPMA – 2-hydroxypropyl methacrylate.

Beyond light, materials sensitive to other external stimuli have also reached clinical evaluation. These include the temperature-sensitive liposomal doxorubicin product ThermoDox,^31^ with several clinical trials ongoing in combination with microwave irradiation, focused ultrasound, and radiation therapy,^31^ and materials responsive to ultrasound, magnetic fields, or radiation. The latter three stimuli have been proportionally less explored than light for therapeutic purposes, but have become increasingly relevant in recent years, especially in combination with nano- and microtechnology (Fig. 1). Prominent examples include ultrasound-responsive microbubbles,^13^ used as contrast agents for clinical ultrasound imaging since the 1990s and investigated for therapeutic purposes from the 2000s onwards;^13,32^ SPION formulations like NanoTherm, clinically approved for the treatment of brain tumors since 2010;^33–35^ and radiation-enhancing hafnium (Hf) oxide nanoparticles like Hensify, approved in 2019 for the treatment of locally-advanced soft tissue sarcoma.^3^

Besides external stimuli, materials responding to internal triggers such as pH,^15,16^ redox,^17^ and enzymatic processes^16,18^ are also extensively developed. Many of these nano- or microparticle systems are designed to deliver small-molecule API in response to different and, ideally, specific (patho)physiological conditions, including high levels of cathepsins (enzyme)^16,18,36^ or glutathione (redox),^17^ or low pH.^15^ Prominent clinical successes include antibody–drug conjugates (ADC), with at least 11 out of 14 products on the market leveraging internal stimuli, such as the pH-sensitive ADC Mylotarg and Trodelvy, and the enzyme-cleavable Enhertu and Tivdak.^16^ The global ADC market size accounted for USD 11.43 billion in 2024 and is predicted to triple by 2034.^37^ Instead, other widely explored systems sensitive to internal stimuli like anticancer polymer–drug conjugates have so far failed to reach FDA/EMA approval.^38^

Despite the extensive research efforts dedicated to the development of stimuli-responsive (nano)materials, many of which have shown high preclinical promise, their clinical translation has been hampered by several challenges. In addition to the added design complexity associated to the stimuli-responsiveness nature, which can complicate scale-up manufacturing and regulatory standardization, other translational aspects such as the lack of specific device availability in many hospitals, the (logistical) complexity of integrating such treatments into standard regimens, and the high associated costs of some devices to generate stimuli like magnetic fields, have also contributed to limiting their clinical impact.

Here, we aim to examine the current landscape of stimuli-responsive materials for medicinal applications, with a particular focus on externally activated nano- and microscale therapeutics and on their translational potential *via* scientometric analysis of existing preclinical and clinical data (Fig. 1). By identifying the most frequently studied and clinically tested stimuli-responsive material platforms for therapeutic purposes, and by analyzing their design principles, mechanisms of action, and specific pharmaceutical and clinical challenges, we provide insights into the developmental trajectories and translational bottlenecks associated with each stimulus type, and we propose strategies to promote their translation and maximize clinical impact.

## 2. Design and preclinical landscape of stimuli-responsive materials

Stimuli-responsive therapeutics rely on activatable small molecules or functional units,^5,9^ which can also be integrated into nano- or microscale platforms.^7,19,39^ They are structurally designed to contain a built-in trigger—such as a π-electron system,^5,14^ transition metal center,^5,40^ labile chemical bond,^41^ or self-assembling/disassembling motif^7,39^—that undergoes a predictable and controlled transition upon exposure to a source of energy (stimulus). Depending on the molecular design, this change can be reversible^5^ or irreversible,^41^ and result in the activation of a range of actions, including structural reorganization,^7,39^ molecular excitations (*e.g.*, through interactions with singlet–triplet excited state),^5^ (metal) catalytic activity,^42^ or payload (API) release.^16,41^ Altogether, these features can be exploited to spatiotemporally control biological and therapeutic effects, minimizing off-target toxicity and enhancing treatment efficacy.^22^ These processes are ultimately governed by the interaction between the applied energy source and specific molecular or material features, which define the mechanism of action for each stimulus modality.

The most relevant classes of stimuli-responsive small molecules consist of fluorescent dyes^5,14^ and, to a lesser extent, also (chemotherapeutic) prodrugs.^9^ These structures can be used alone or formulated into nano-/microscale carriers such as liposomes,^7^ lipid nanoparticles,^43^ micelles,^44,45^ hydrogels,^39^ synthetic polymers,^46,47^ polycarbohydrates (*e.g.*, chitosan and dextran),^48,49^ antibodies and ADC,^16,19^ and (inorganic) solid nanoparticles.^50,51^ There are also source-specific nano- and micro-materials that display intrinsic responses upon the application of a stimulus due to specific compositions and sizes, or the incorporation of reactive functional units in their structure. Examples of these include quantum dots (light),^52^ microbubbles^53,54^ and nanobubbles^55^ (ultrasound), and metal(loid)-based nanoparticles based on gold^56,57^ (light), iron (SPION;^10,11,58^ magnetic field), and hafnium,^2,3^ gadolinium^59^ or boron-10^60^ (radiation).

Five major external stimuli can be categorized, each representing a distinct energy source that acts as a driving force to activate the stimuli-responsive material and trigger biological effects. These stimuli include light, temperature, ultrasound, radiation and magnetic field. In parallel, endogenous stimuli such as changes in pH, redox potential and enzymatic levels are also relevant in the design of therapeutic materials. Some external stimuli have already led to the development of distinct clinical strategies. For instance, light-activated therapies encompass PDT,^4^ PTT^28^ and PIT;^29^ and ultrasound-based therapies include sonodynamic therapy (SDT),^14^ but also sonopermeation approaches, which combine microbubbles and ultrasound to enhance chemo-, nano-, and immunotherapies.^53,61^ Radiotherapy efficiency can be enhanced by radiation-responsive materials,^2,59^ and magnetic-induced hyperthermia (MIH) harnesses magnetic nanoparticles to generate localized heat upon exposure to an alternating magnetic field, leading to targeted tissue ablation.^10,11^

**Fig. 2:**
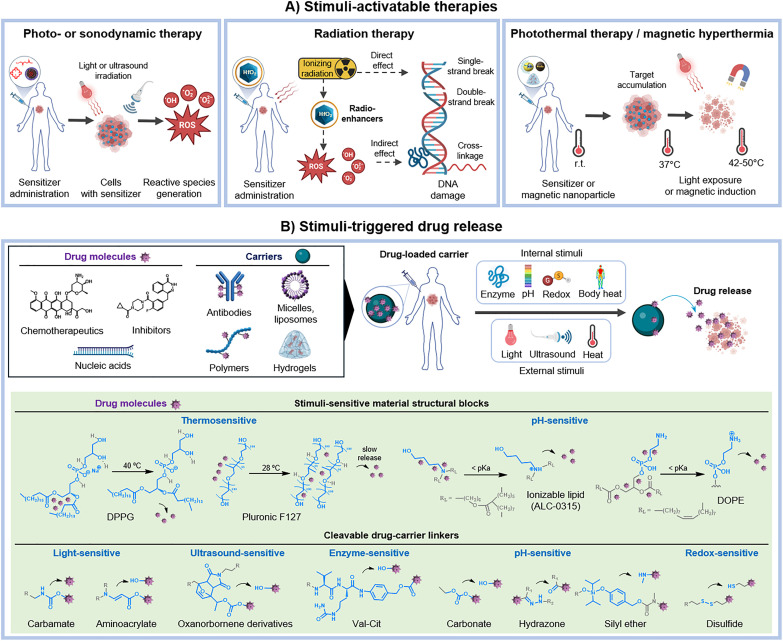
Modes of action of stimuli-responsive materials for therapeutic purposes. (A) *Stimuli-activatable therapies.* Stimuli-responsive materials can induce direct biological and therapeutic effects mediated by the interaction of the material with the external stimuli. These mostly include radical and reactive oxygen species (ROS) generation (for PDT, PIT, SDT and RT), which induce biomolecule/DNA damage and oxidative stress, radiation-induced DNA damage (for RT), and heat-mediated cell ablation (for PTT and MIH). (B) *Stimuli-triggered drug release.* Stimuli-responsive materials can act as passive carrier systems, delivering active payloads (from small chemotherapeutics to macromolecules like proteins and nucleic acids) upon external or internal stimuli. This process can be promoted (1) by inducing changes in the (nano)carrier structure (*e.g.*, swelling or disassembling of hydrogels, liposomes or micelles), and (2) cleavage of the linker between the drug and the carrier (*e.g.*, in antibody- and polymer–drug conjugates). *Abbreviations*: PDT – photodynamic therapy, PIT – photoimmunotherapy, SDT – sonodynamic therapy, RT – radiation therapy, PTT – photothermal therapy, MIH – magnetic-induced hyperthermia, DPPG – dipalmitoylphosphatidylglycerol, DOPE – dioleoyl phosphatidylethanolamine.

In general, the stimuli-responsiveness capability of the materials can be therapeutically exploited in two different ways: either (a) directly inducing biological effects to cells and tissues after external stimulus application (Fig. 2A), or (b) acting as a passive carrier system and releasing active payloads (*e.g.*, chemotherapeutics) upon internal or external stimuli (Fig. 2B). Regarding the former, the mechanism of action can vary slightly depending on the source of energy (Fig. 2A), encompassing (i) the production of cell-damaging radicals like reactive oxygen species (ROS) (for PDT,^4^ PIT,^29^ SDT^14^ and RT^62,63^) and radiation-induced nucleic acid damage (for RT^63^); and (ii) the conversion of the source energy into heat by the material (for PTT^56^ and MIH^64^), subsequently leading to biomolecule and tissue ablation. These mechanisms not only promote cell death at the target site, but can also result in the induction of systemic immune responses, with potential for immunotherapy applications.^65^

Regarding stimuli-triggered drug release (Fig. 2B), the stimulus does not directly activate the therapeutic agent itself, but it induces structural and chemical changes in the carrier material, promoting the delivery of the active payloads. This can involve assembling/disassembling of the delivery platform (*e.g.*, as observed in hydrogels,^39^ liposomes^7^ and micelles^44^), or cleavage of a linker between the carrier material and the drug (*e.g.*, in ADC^41^ and polymer conjugates^46,66^). In contrast to external stimuli-activated therapies, which can directly mediate biological effects through the material itself, stimuli-triggered drug release only facilitates payload delivery, enabling the therapeutic agent (API) to act independently at the target site.

**Fig. 3:**
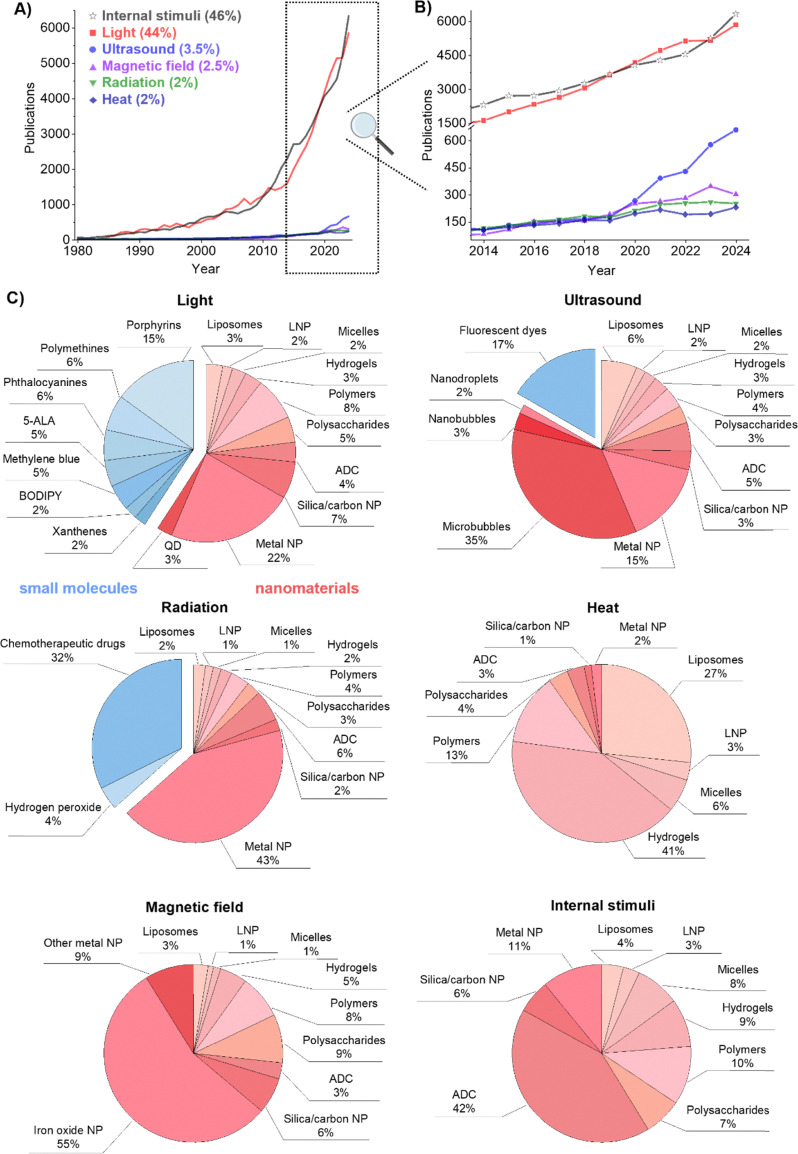
Landscape of stimuli-responsive therapeutics in preclinical research. (A) and (B) Number of research publications over the years, starting from 1980 until 2024 (a total of 131199 publications) (A), and zoom-in between 2014 and 2024 (91691 publications) (B). (C) Distribution of the scope and type of materials responsive to light, ultrasound, magnetic field, radiation, temperature/heat, and internal stimuli (pH, enzymatic levels and redox activity) in the last decade. The blue color corresponds to small molecules, and the red color to nano-/microscale materials. Data were obtained in October 2025, from Digital Science's Dimensions platform, available at https://app.dimensions.ai. Keywords used for the search are shown in Scheme S1. *Abbreviations*: LNP – lipid nanoparticles, ADC – antibody–drug conjugates, NP – nanoparticles, QD – quantum dots, 5-ALA – 5-aminolevulinic acid.

To understand how the different stimuli have impacted materials research and development for therapeutic applications over the last years, we first analyzed the landscape of related publications between 1980 and 2024 (Fig. 3). Five main external stimuli were considered (light, temperature/heat, ultrasound, radiation and magnetic field), and three common internal stimuli (pH, enzyme levels and redox activity). This analysis covered small molecules and nano- and micro-scale structures, and involved 91691 publications and 1083 trials, by using the keyword search shown in Scheme S1.

Based on the data presented in Fig. 3A and B, light- and internal stimuli-responsive materials have thus far dominated the field (46% and 44%, respectively), together accounting for approximately 90% of all the publications on stimuli-responsive therapeutics in the last 40 years. Both categories have exhibited comparable growth trajectories, with an exponential increase over the last two decades, reaching about 6000 publications each only in 2024.

Comparatively, the other stimulus modalities have received significantly less attention for therapeutic purposes, although some emerging trends are noteworthy. Publications on ultrasound-responsive therapeutic materials have tripled over the past five years, reaching about 600 publications. The research interest in magnetic field-responsive therapeutics has increased about 5-fold in the last decade (∼300 publications, despite the slight recession observed in 2024). The research growth in the latter case is likely encouraged by the approvals of several SPION formulations during the early 1990s and 2000s for the treatment of anemia and imaging,^58^ and, quite relevantly, by the approval of the magnetic-responsive SPION product NanoTherm in 2010 as a medical device for the treatment of glioblastoma.^35^ On the other hand, radiation therapy-potentiation using nanoscale radioenhancers like Hensify^3^ or small-molecule chemotherapeutic agents like cisplatin, which have been reported to radiosensitize cells,^9,67^ has shown a steady growth reaching ∼250 publications annually in the 2020s. The attention to temperature-responsive materials peaked in 2021, but has since stagnate (∼200 publications annually), which might be partly influenced by the failed phase III clinical trials of the thermosensitive liposomal doxorubicin formulation ThermoDox in 2013 (HEAT trial),^68^ and later in 2020 (intermediate results of OPTIMA trial).^69^

We then analyzed the scope and type of materials explored for each stimulus in preclinical research (Fig. 3C). The searches included small molecules (*e.g.*, fluorescent dyes and (chemo)therapeutic agents) and relevant nano- and micromaterial classes (liposomes, lipid nanoparticles, micelles, hydrogels, polymers, polysaccharides, ADC, and non-metal and metal solid nanoparticles). As a general observation, the impact of nanotechnology and microtechnology is evident from the data. In light-responsive and radiation-responsive materials, this accounts for more than half of the retrieved publications (59% and 64%, respectively), and it becomes predominant in ultrasound-responsive materials (83%), and even more in heat-, magnetic field-, and internal stimuli-responsive materials. Regarding the latter, it is important to note that in addition to the impact of nanotechnology in the development of internal stimuli-responsive therapeutics (*e.g.*, antibody- and polymer–drug conjugates), small molecule prodrugs responsive to pH, enzyme levels or redox have also been widely explored, with several clinical products like ixazomib citrate (cancer), pretomanid (tuberculosis), or omidenepag isopropyl (glaucoma and hypertension).^70,71^ However, many of these prodrugs are insoluble or present suboptimal pharmacokinetic properties, thus often being evaluated preclinically together with nanoscale delivery systems. This feature strongly influenced the results obtained using our keyword search query (Scheme S1), making it difficult to accurately estimate the preclinical landscape and research relevance of only internally activatable small-molecule prodrugs (with many of these reported as part of nanoparticle formulations).

### 2.1. Light-responsive materials

Light-responsive systems primarily rely on photon absorption by chromophores, leading (1) to excited-state reactions that generate ROS (PDT/PIT)^4^ or (2) to non-radiative relaxation processes that convert light into heat (PTT).^56^ Small molecules are highly relevant in the design of light-responsive materials, representing close to 40% of the total number of publications in the field, as compared to the 59% covered by nanomaterials. Classical photosensitizers remain prominent for PDT, with π electron-conjugated small molecules like porphyrins, phthalocyanines, 5-aminolevulinic acid (5-ALA, a naturally occurring porphyrin precursor in the human body), Methylene Blue, and xanthene derivatives (*e.g.*, Rose Bengal) collectively accounting for about 33% of the publications on light-responsive materials (Fig. 3C). Regarding nanomaterial classes, metal nanoparticles represent 22% of the total number of publications on light-activated materials (Fig. 3C), with gold being among the most studied metals. Gold nanoparticles are known to play a central role in PTT, relying on heat generation through plasmonic resonance excitation for targeted cell ablation.^56,57^ Liposomes, lipid nanoparticles, natural (polysaccharides) and synthetic polymers, and hydrogels, present in about 25% of the total number of publications on light-sensitive materials, are widely used as carrier materials of light-responsive molecules like porphyrins, Methylene Blue or phthalocyanines for both PTT and PDT. Photosensitizer-carrying ADC have given rise to the PIT field,^72^ which combines the advantages of immunotherapy with PDT for synergistic effects.^73^ Although PIT is only present in 4% of the total publications on light-responsive therapeutic systems, its interest has been growing over the last years, partly driven by the preclinical promise observed with several ADC bearing IR700 and 2ICy7 dyes.^72,74^ In particular, the outcomes of the clinical trial involving the IR700-bearing antibody (discussed in Section 3) can lead to further developments in the field of light-sensitive ADC.

Despite the promising preclinical data, light penetration in human tissue is limited to a depth of approximately 1.5 cm, which significantly restricts the clinical use of light-triggered therapies to superficial conditions (*e.g.*, actinic keratosis).^75^ Thus, there has been a growing interest towards the development of new materials sensitive to near-infrared (NIR) light, which exhibit deeper tissue penetration capabilities, particularly to NIR-I (700–900 nm wavelength) and NIR-II (900–1700 nm wavelength) optical windows.^6^ These materials could act in more biologically transparent regions, effectively reaching deeper-seated tissues (up to 4 cm).^76^ Such materials include small molecules like polymethines (6%; such as iodinated cyanines for bacterial eradication^77^ and cancer treatment^74^), BODIPY,^78^ or metal coordination complexes (*e.g.*, ruthenium compounds), as well as donor–acceptor–donor polymers,^79^ carbon nanoparticles,^80^ quantum dots,^81,82^ and metal nanoparticles.^83^ Despite the extensive preclinical development in NIR-I- and NIR-II-activatable therapeutics, only the ruthenium complex TLD1433 and the IR700-bearing antibody have reached clinical trials to date.^84^

### 2.2. Ultrasound-responsive materials

While phototherapies rely on light sources (*i.e.*, electromagnetic waves), ultrasound renders waves that are mechanical by their nature. Hence, they can enable deeper tissue penetration, up to 10 cm, making it well-suited for targeting and treating deep-seated lesions.^85^ However, both sound scattering and cavitation control must be considered to fully realize its potential.^86,87^ Depending on the parameters (intensity, frequency and acoustic pressure), ultrasound can induce thermal or mechanical effects, the latter *via* cavitation.^86,87^ To promote mechanical effects, microbubbles, which are gas-filled vesicles of typically 1–5 µm in diameter, are particularly effective and represent the most extensively studied material in the field,^88^ with ∼35% of related publications. Originally developed as contrast agents for the diagnosis of cardiac and hepatic lesions, microbubbles oscillate in response to ultrasound.^87^ These oscillations not only enhance imaging signals but can also “massage” vascular endothelium, hence, temporarily opening biological barriers like the blood–brain barrier and tumor vasculature through sonoporation or sonopermeation.^89,90^ Since the 2000s, microbubble-mediated therapy has sparked intense preclinical research, reaching clinical evaluation for the treatment of glioblastoma,^91,92^ inoperable pancreatic cancer^93^ and neurodegenerative diseases.^94^

Lipid-coated microbubbles such as SonoVue (Bracco) and Definity (Lantheus) are already employed for intravenous co-administration with various therapeutics in clinical trials, depending on the cancer type and study protocol. However, microbubbles with thicker shell, coated with albumin (Albunex, Optison) or synthetic polymers like poly(butyl cyanoacrylate), offer higher payload capacity.^47,95^ In this setup, microbubbles serve as cavitation nuclei, while the loaded agents provide additional targeting or therapeutic functions. Since microbubbles are confined to the vascular compartment and are rapidly cleared within a few minutes, they can offer a transient and localized delivery platform once activated with ultrasound.^96,97^ Therefore, loaded microbubbles may improve the safety of drugs that cause off-target toxicity upon systemic administration. Nanobubbles^98^ and phase-change nanodroplets^99^ (jointly contributing to 5% of the publications) are currently being investigated as alternatives to microbubbles, aiming to achieve longer circulation times while preserving cavitation responsiveness and enabling deeper target-tissue penetration.

Although far less investigated than microbubbles, other nanoparticle platforms like liposomes can also be engineered to selectively respond to ultrasound by introducing gas in their cavities or excipients in the formulation that alter the acoustic properties of the liposome core medium.^100^ In particular, the latter has recently been demonstrated to be effectively activated acoustically, enabling controlled and site-targeted drug delivery and neuromodulation.^101^

Finally, small-molecule fluorescent dyes, accounting for 17% of publications in the field, are commonly employed in SDT.^14^ These dyes are mainly based on porphyrins and their precursor 5-ALA, Methylene Blue, and xanthenes like Rose Bengal. Recently, heptamethine cyanines have also shown promising preclinical outcomes in SDT.^102^ While the exact mechanisms of SDT remain not fully understood, it is hypothesized that these molecules follow a reaction cascade similar to PDT, locally generating ROS in deep-seated tissues, beyond the reach of conventional light-based approaches.^103^

### 2.3. Radiation-responsive materials

Radiation-responsive systems primarily function by absorbing ionizing radiation and generating secondary electrons and reactive species, which enhance DNA damage and oxidative stress in tissues. Metal nanoparticles, which account for about 43% of all publications in this category (Fig. 3C), are the most extensively studied materials. Elements with high atomic (Z) number, such as gold, hafnium, and gadolinium, exhibit strong photoelectric absorption and Auger electrons, producing secondary electrons, which can interact with surrounding water and biomolecules to generate ROS and induce oxidative stress, amplifying radiation-induced DNA damage, and consequently promoting cell death.^2,3,56,59^ However, under clinically relevant irradiation conditions in the megavoltage energy range, where Compton scattering dominates and differences in photon attenuation become weakly dependent on atomic number, the therapeutic efficacy of these materials cannot be attributed solely to enhanced photon absorption. Instead, as the radiation beam propagates through the tissues, it undergoes spectral degradation (beam softening), leading to the generation of low-energy photons and secondary electrons that interact more efficiently with high-Z elements.^104,105^ Their radiosensitizing effects therefore likely involve a combination of physical, chemical, and biological mechanisms, including interactions with these secondary radiation components that increase local secondary electron production and promote electron cascades yielding a higher proportion of short-range, low-energy electrons. These processes result in highly localized energy deposition and enhanced radiolysis-mediated ROS generation, which can amplify downstream biological responses, such as DNA damage complexity, increasing radiation effectiveness.^106,107^ Clinically relevant examples include AGuIX and Hensify radio-enhancing nanoparticles for cancer therapy, which will be further discussed in Section 3. Among non-metal-based nanoparticles, boron-10 has shown particularly promising radiosensitizing properties. Although mechanistically distinct from the previously mentioned radioenhancers, boron-10 enables targeted radiation dose delivery *via* neutron capture, which has also been reported to activate the immune system and improve treatment outcomes in combination with immunotherapy.^108,109^

Interestingly, some chemotherapeutic drugs, such as cisplatin, carboplatin, fluorouracil, gemcitabine and capecitabine, have also been demonstrated to potentiate radiation therapy performance,^9,110^ despite not being directly responsive to radiation or designed to primarily act as radiosensitizers. Although in most cases the interplay between DNA, nuclear proteins, and drugs under radiation remains unclear, studies suggest that cisplatin binding to DNA increases its reactivity towards near-zero-eV (∼0.5 eV) electrons, which are abundant during radiotherapy, preventing effective DNA-damage repair after ionizing radiation.^111^ Similarly, there are other small molecules, such as Methylene Blue,^112^ vitamin D,^113^ and DNA repair inhibitors like Latexin^114^ and mTOR,^115^ also reported to potentiate radiotherapy. However, data in these cases are scarce, mechanisms are still unclear^113^ or the clinical significance is compromised.^115^ Fluorescent dyes are also explored as radiopotentiating molecules in radiodynamic therapy (RDT), with focus on porphyrin derivatives. While the underlying mechanisms remain poorly understood, several radiation-induced activation pathways have been proposed, including Cherenkov radiation, interactions with secondary electrons, and biochemical or biomolecular sensitization, ultimately contributing to increased tumor susceptibility to irradiation.^116^

Antibodies and ADC (∼6%, Fig. 3C) are being also explored in chemoradiotherapy settings, either similarly to conventional chemotherapeutic drugs^117^ or in combination therapy regimens, such as immune checkpoint inhibitors co-administered with hafnium oxide nanoparticles.^118^

It is well known that the hypoxic tumor microenvironment limits ROS-related therapeutic mechanisms, reducing (radio)therapy efficacy.^119^ A direct strategy to counter this involves oxygen switches,^120^ such as hydrogen peroxide (which is part of 4% of the total number of publications in radiation-sensitive materials, as indicated in Fig. 3C). Hydrogen peroxide can be, for example, (co-)formulated with sodium hyaluronate for intra-tumoral injections to oxygenate hypoxic regions and enhance ROS generation upon radiation.^62^

### 2.4. Magnetic field-responsive materials

Magnetic field-responsive materials operate through magnetic relaxation processes that convert alternating magnetic field energy into heat, enabling localized hyperthermia or triggered drug release. They primarily rely on iron oxide-based nanoparticles (55% of MRI-related therapeutic applications, Fig. 3C), mostly magnetite (Fe_3_O_4_).^58^ Superparamagnetic iron oxide nanoparticles (SPION), extensively explored for iron-deficiency anemia treatment and magnetic resonance imaging applications,^58^ can generate heat under the application of an alternating magnetic field, making them also ideal candidates for magnetic hyperthermia and magnetically guided drug delivery.^11^ Surface modification of SPION enables the modulation of colloidal stability and biocompatibility (*e.g.*, by functionalizing them with biocompatible polymers like carbohydrates), while magneto-thermal conversion efficiency is primarily determined by the properties of the SPION core. This efficiency, often measured by the specific absorption rate, depends on magnetic parameters such as saturation magnetization, magnetic anisotropy, and relaxation mechanisms (*e.g.*, Néel and Brownian).^10,121^ High specific absorption rate values are typically achieved by optimizing particle core size (below 30 nm), shape, and crystalline structure of SPION to enhance magnetic responses.^122^

Mixed ferrites (MFe_2_O_4_, where M stands for cobalt, manganese, nickel or zinc) are also investigated (∼9% of publications in the field, Fig. 3C) to improve magnetic performance over conventional magnetite (Fe_3_O_4_), with properties varying based on the incorporated dopant.^123–125^ For instance, manganese doping enhances magnetic anisotropy and saturation magnetization, thereby improving thermal response under an alternating magnetic field.^124^ Cobalt doping, on the other hand, significantly enhances magnetic anisotropy and coercivity, enabling more efficient heat generation *via* Néel relaxation.^125^ These dopants help align the magnetic relaxation time with the field frequency used in clinical hyperthermia, maximizing heat production while maintaining biocompatibility when administered at clinically relevant concentrations.^10,123–125^

In addition to these, nanostructures like synthetic polymers and polysaccharides are also relevant in magnetically activated materials (17% of publications in the field, Fig. 3C). They often act as coating agents for SPION, preventing aggregation and improving their colloidal stability, biocompatibility, and resistance to oxidation.^126,127^ Finally, several works have shown how SPION can also be integrated in hydrogels, liposomes or micelles, resulting in hybrid (nano-)constructs and facilitating their thermal-mediated reorganization or destruction when a magnetic field is applied.^128–130^ Finally, hybrid materials integrating SPIONs into stents or vascular grafts are gaining attention for their theranostic potential. In such systems, SPIONs not only enable real-time tracking *via* MRI and magnetically guided localization but also serve as active therapeutic agents, either through magnetic hyperthermia or by facilitating externally triggered drug release. This multifunctional approach has shown promise in enhancing both patient-centered treatment precision and efficacy in vascular applications.^131^

### 2.5. Temperature-responsive materials

Conceptually, heat-responsive systems do not rely on direct energy–material interactions, but on temperature thresholds that trigger phase transitions or structural changes in the carrier or linker, controlling drug release. Unlike the previous external stimuli, temperature itself cannot be categorized as a direct source of energy, but rather as the mediator triggering the therapeutic outcome. Typically, electromagnetic or mechanical waves (*i.e.*, external stimuli such as light, ultrasound, magnetic field and radiation) are exploited to locally generate heat and to either directly ablate target lesions or trigger drug release. Instead, we here focus specifically on thermal-triggered drug release.^31,132^ As shown in the research landscape analysis in Fig. 3C, hydrogels (41%), liposomes (27%), and polymers (13%) dominate the research on heat-responsive materials, highlighting their potential for precise, temperature-controlled drug release.^31,133,134^

These materials exhibit a sharp thermal responsiveness due to phase transitions or conformational rearrangements near their lower critical solution temperature. For instance, poly(*N*-isopropylacrylamide) undergoes a coil-to-globule transition around 32 °C, resulting in abrupt changes in hydrophilicity and volume that can trigger drug release from nanocarriers.^132^ Polyethylene oxide-based non-ionic surfactants, Pluronics or Poloxamers, exhibit sharp sol–gel transition at 28–29 °C.^135^ Similarly, thermosensitive liposomes destabilize near 42 °C as lipid bilayers transition from a gel to liquid-crystalline phase (*e.g.*, in the cancer nanomedicine ThermoDox), enhancing permeability and enabling burst release of encapsulated drugs such as doxorubicin.^31^

### 2.6. Internal stimuli-responsive materials

Internal stimuli-responsive therapeutics are engineered to exploit endogenous (patho-)physiological features (such as abnormal changes in pH, redox potential, or enzymatic activity) to achieve controlled drug delivery and therapeutic effects. Among these, antibody–drug conjugates (ADC) dominate the field, accounting for 42% of related publications (Fig. 3C). ADC achieve selective targeting through antibody recognition of disease biomarkers, coupled with linkers that release cytotoxic payloads intracellularly after exposure to a specific internal trigger, such as high enzymatic activity or low pH.^16,19,66^ ADC can also facilitate the so-called bystander effect, a phenomenon in which the drug payload can diffuse into and kill neighboring cells, even those that do not express the target antigen on their surface.^136^ Target antigens of the approved ADC drugs are typically specific proteins overexpressed in cancer cells, including HER2,^137^ Trop2,^138^ nectin-4 and EGFR in solid tumors, and CD19, CD22, CD33, CD30, BCMA and CD79b in hematological malignancies.^16^ Clinically approved examples of ADC include Enhertu (HER2+ target) and Tivdak (tissue factor target), which use cathepsin-sensitive linkers, and Trodelvy (Trop2 target), which relies on acid-cleavable carbamate chemistry.^16,19^

To overcome resistance and safety limitations of conventional ADC, bispecific ADC targeting two epitopes or antigens to enhance internalization and specificity are gaining popularity.^16^ Probody-drug conjugates and bispecific ADC are emerging formats designed to improve tumor selectivity and reduce off-target toxicity. Probody-drug conjugates use a protease-cleavable peptide mask that blocks antigen binding in healthy tissues and is removed in the tumor microenvironment.^139^ Since tumors can develop resistance to cytotoxic drugs, alternative ADC strategies involve conjugating multiple payloads that act *via* distinct mechanisms (*e.g.*, a TOP1 inhibitor targeting DNA replication, and a RNA polymerase II inhibitor blocking transcription), resulting in dual payload ADC, which are recently moving into first oncology clinical trials.^140^ Tumor heterogeneity and antigen loss can further limit ADC efficacy by promoting the outgrowth of antigen-negative subpopulations.^139^ In this context, stimuli-responsive ADC, including cleavable linkers enabling bystander effects and bispecific targeting, may help mitigate such evolutionary selection, although complete prevention of resistance remains challenging.

Beyond ADC, a diverse set of nanocarriers has been developed to respond to internal triggers. Examples of these encompass metal nanoparticles (11%), polymers (10%), hydrogels (9%), micelles (8%), liposomes (4%) and lipid nanoparticles (3%) (Fig. 3C). In these systems, the drug can be either directly conjugated to the carrier *via* a cleavable linker^7,46,66^ or non-covalently encapsulated.^7,39,44,46^ Three main stimuli are explored (changes in pH, redox and enzyme levels), and two main strategies are followed to develop internally triggerable therapeutics. These include the incorporation of stimuli-sensitive functional groups into the structure of the carrier, destabilizing it upon stimulus application and promoting drug release (particularly when the drug is non-covalently encapsulated), or conjugating the drug to the carrier *via* a sensitive, cleavable bond.

Redox-sensitive nanocarriers commonly exploit disulfide bonds that cleave in reductive, glutathione-rich environments, for instance, in tumor cells^17,46,66,141^ or inflammation sites. Instead, pH-responsive systems can be stratified by their activation site, namely, the slightly acidic tumor microenvironment (pH ∼ 6.5–7.2), endosomes (pH ∼ 5.5–6.8) or lysosomes (pH ∼ 4.5–5.5); each requiring distinct structural adaptations. pH-sensitive liposomes aiming for drug release in the tumor microenvironment can incorporate DOPE lipids (1,2-dioleoyl-*sn*-glycero-3-phosphoethanolamine). These liposomal formulations have already shown preclinical efficacy for doxorubicin delivery due to the destabilization of the liposomal membrane at pH ∼ 6.5 and the subsequent release of the drug.^142^ Lipid nanoparticles, for instance, contain ionizable lipids with tertiary amines (p*K*_a_ ∼ 6.0–6.7) that facilitate endosomal escape after protonation at endosomal pH, together promoting effective intracellular delivery of RNA therapeutics.^143,144^ Acid-labile linkers such as hydrazones^145,146^ and acetals^147^ are also widely used to trigger drug release during lysosomal trafficking.

Enzyme-cleavable nanomaterials, especially those responsive to a cathepsin-cleavable valine–citrulline (Val–Cit) linker,^148^ matrix metalloproteinase-cleavable linkers^149^ or glycosidic bond cleavage,^66,150^ have also shown preclinical and clinical promise. However, the translation of such systems remains minimal compared to ADC. For instance, earlier-generation systems, such as drug-HPMA copolymers conjugates with matrix metalloproteinases-cleavable linkers, underwent clinical testing but failed to progress further,^38^ underscoring the challenge of moving enzyme-responsive designs beyond the proof-of-concept stage. Several reasons can be alluded to, primarily related to the drug-polymer conjugate design and linker choice, as well as the intrinsic heterogeneity of enzyme levels in biological systems.

Finally, and albeit not traditionally considered as “stimuli-responsive therapeutics”, it is worth noting that several nanomedicines that have been already approved for clinical use are in fact responsive to internal stimuli. Besides the abovementioned pH-dependency of lipid nanoparticles for efficient encapsulation and intracellular delivery of RNA (*e.g.*, in Onpattro and Comirnaty),^143^ liposomes and polymeric micelles (*e.g.*, Doxil, DaunoXome, Nanoxel) also exploit the acidic pH of lysosomes to trigger drug release *via* decomposition of the shell.^151^ Together, these examples underscore that internal stimuli-responsiveness can be embedded in clinically successful formulations when paired with a clear biological rationale and adequate material design.

## 3. Translational status of stimuli-responsive therapeutics

**Fig. 4:**
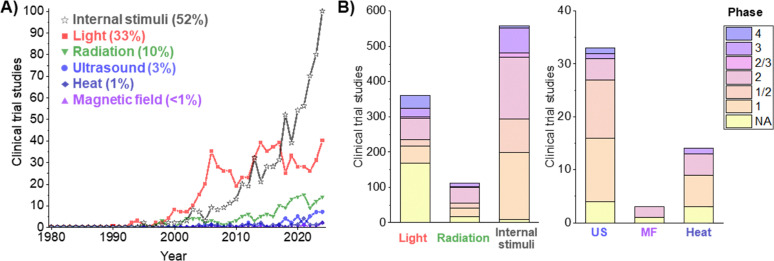
Evolution of clinical trials with stimuli-responsive therapeutics. (A) Number of clinical trial cases starting from 1980 until 2024 for the different classes of stimuli-responsive therapeutics. (B) Distribution of clinical trial phases of the studies between 2014 and 2024 (1083 trials). Data were obtained in October 2025, from the online database of clinical research studies, available at https://clinicaltrials.gov/. The data were analyzed and manually sorted out to exclude false positive cases, unrelated to stimuli-responsive therapeutics (*e.g.*, devices or only diagnostic interventions). *Abbreviations*: US – ultrasound, MF – magnetic field.

To assess the clinical relevance of these materials, we analyzed clinical trials involving stimuli-responsive therapeutics from the 1980s until 2024 (Fig. 4). For that, we used the same keyword strategy (Scheme S1) applied in the research landscape analysis (Fig. 3). Consistent with the trends in preclinical studies, materials responsive to internal stimuli and light dominate the clinical landscape (52% and 33%, respectively), together accounting for about 85% of all the clinical trials in this space. Several of the corresponding product candidates have progressed to late-stage trials, including phase III and IV (Table 1). Radiation-responsive materials, primarily involving radiosensitizers, account for about 10% of clinical trials, while ultrasound-responsive systems for therapeutic purposes represent ∼3% (although both include studies in advanced clinical phases, *e.g.*, NCT05317858, NCT04667494, NCT04892173). In contrast, heat- and magnetic-field-responsive materials have remained clinically underrepresented over the last four decades.

**Table 1:** Translational status of selected stimuli-responsive therapeutics evaluated in clinical trials

Stimulus	Therapeutic/material (Brand name)		Active component^a^	Therapy	Indication	Clinical trial phase	Clinical trial number
Light	Small molecule	Ru(ii) coordination complex (Ruvidar)^b^	—	Photodynamic therapy	Non-muscle invasive bladder cancer	II	NCT03945162
		5-aminolevulinic acid;5-ALA^b^	—	Photodynamic therapy	Actinic keratosis	IV	NCT05359419, NCT03642535
		Hemoporfin^b^	—	Photodynamic therapy	Port-wine stain	IV	NCT04106258
		Methylene Blue^b^	—	Photodynamic therapy	Peri-implant disease	IV	NCT04187053
		Porphyrin derivative^b^	—	Photodynamic therapy	Bile duct cancer	IV	NCT05551299
		Indocyanine Green^b^	—	Photodynamic therapy	Periodontitis	II/III IV	NCT04857346 NCT04964167
	Nano/Micro	IR700-cetuximab^b^ (ASP-1929)	IR700 (Phthalocyanine derivative)	Photoimmunotherapy	Head and neck cancer	III	NCT03769506
		Visudyne^b^	Verteporfin	Photodynamic therapy	Pancreatic cancer	II	NCT03033225
		Indocyanine Green-chitosan nanoparticles^b^	Indocyanine Green	Photodynamic therapy	Periodontitis	N/A	NCT06523244
		5-ALA-loaded hydrogel^b^	5-aminolevulinic acid (5-ALA)	Photodynamic therapy	Wound healing	N/A	NCT06445699
		Gold–silica nanoparticles (AuroShell)^b^	Gold–silica nanoparticles	Photothermal therapy	Prostate cancer	I/II	NCT04240639
		Gold-silver-cuprous oxide-containing nanogel^b^	Gold-silver-cuprous oxide composite	Photothermal therapy	Microbial keratitis	I	NCT05268718
		Methylene Blue-loaded chlorhexidine gel^b^	Methylene Blue	Photodynamic therapy	Periodontitis	N/A	NCT06469294
Ultrasound	Small molecule	5-Aminolevulinic acid^b^	—	Sonodynamic therapy	Glioma	II	NCT04845919
		Sinoporphyrin^b^	—	Sonodynamic therapy	Atherosclerosis	I/II	NCT03457662
	Nano/Micro	Microbubbles (Definity)^b^	Perflutren (C_3_F_8_)	Sonopermeation-mediated delivery of carboplatin	Glioblastoma	III	NCT05902169
				Sonopermeation-mediated delivery of pembrolizumab	Brain metastases of non-small cell lung cancer	III	NCT05317858
		Microbubbles (SonoVue)^b^	Sulphur hexafluoride (SF_6_)	Sonopermeation-mediated delivery of FOLFIRINOX	Pancreatic ductal adenocarcinoma	II	NCT04146441
				Sonopermeation-mediated delivery of bevacizumab	Glioblastoma	III	NCT06496971
Radiation	Small molecule	Hydrogen peroxide^c^ (KORTUC)	—	Radiotherapy	Breast cancer	II	NCT03946202
		5-aminolevulinic acid^c^	—	Radiodynamic therapy	Glioblastoma	I/II	NCT05590689
		Cisplatin^c^^d^	—	Chemo-radiotherapy (plus pembrolizumab)	Head and neck cancer	III	NCT03040999
		Cisplatin and paclitaxel^c^^d^	—	Chemo-radiotherapy	Non-small cell lung cancer	III	NCT06545747
		Cisplatin and nimorazole^c^^d^	—	Chemo-radiotherapy	Head and neck cancer	III	NCT01880359
		Cisplatin and gemcitabine^c^^d^	—	Chemo-radiotherapy (plus sintilimab)	Nasopharyngeal cancer	III	NCT03700476
		Pirfenidone^c^^d^	—	Radiotherapy	Head and neck squamous cell carcinoma	II	NCT06142318
		Ropidoxuridine^c^	—	Radiotherapy	Glioblastoma	II	NCT06359379
		Tranilast^c^^d^	—	Radiotherapy	Nasopharyngeal carcinoma	II	NCT05626829
	Nano/Micro	Hafnium(iv) oxide nanoparticles (Hensify; NBTXR3)^b^	Hafnium(IV) oxide	Radiotherapy	Head and neck squamous cell carcinoma	III	NCT04892173
					Soft tissue sarcoma	II/III	NCT02379845
		Gadolinium nanoparticles (AGuIX)^b^	Gadolinium(III) -DOTAGA complex	Radiotherapy	Brain metastases	II	NCT03818386
		Metal–organic framework (RiMO-301)^b^	Hafnium complex	Radiotherapy	Advanced tumors	I	NCT03444714
		Fluorocarbon emulsion (NVX-108)^d^	Dodecafluoropentane (C_5_F_12_)	Radiotherapy	Glioblastoma	I	NCT02189109
Magnetic field	Nano/Micro	SPION (NanoTherm)^b^	Fe_3_O_4_ nanoparticles	Magnetic hyperthermia	Focal prostate cancer	IIB	NCT05010759
					Glioblastoma	N/A	NCT06271421
		SPION^b^	Fe_3_O_4_ nanoparticles	Magnetic hyperthermia	Osteosarcoma	I	NCT04316091
		SPION^b^ (NTT agent)	γ-Fe_2_O_3_ nanoparticles	Magnetic hyperthermia	Pancreatic ductal adenocarcinoma	N/A	Not assigned
Heat	Nano/Micro	Liposomal doxorubicin (ThermoDox)	Doxorubicin^c^	Chemotherapy	Hepatocellular carcinoma	III	NCT02112656
		Mitomycin C-loaded hydrogel (UGN-103)	Mitomycin C^c^	Chemotherapy	Urothelial cancer	III	NCT06331299
		5-fluorouracil-loaded hydrogel	5-Fluorouracil^c^	Chemotherapy	Colorectal cancer	II	NCT06385418
		Nitazoxanide-loaded Hydrogel	Nitazoxanide^c^	Antimicrobial therapy	Periodontitis	II	NCT04768530
		Octenidine-loaded hydrogel	Octenidine^c^	Antimicrobial therapy	Periodontitis	N/A	NCT06522438
Internal stimuli	Nano/Micro	Mirvetuximab soravtansine (MIRV)	DM4^c^	Chemotherapy (redox-sensitive)	Ovarian, peritoneal, fallopian tube cancer	III	NCT04209855
		Bispecific antibody–drug conjugate (BL-B01D1)	Toxin Ed-04^c^	Chemotherapy (enzyme-sensitive)	Advanced solid tumors	I	NCT05194982
		Probody-drug conjugate (CX-2029)	Monomethyl auristatin E^c^	Chemotherapy (enzyme-sensitive)	Advanced solid tumors	I/II	NCT03543813
		Nimesulide-loaded polymeric micelles (CA102N)	Nimesulide^c^	Chemotherapy (pH-sensitive)	Colorectal cancer	II	NCT06039202
		Epirubicin-loaded polymeric micelles (NC-6300)	Epirubicin^c^	Chemotherapy (lysosomal degradation)	Advanced solid tumors, sarcomas	I/II	NCT03168061

a In the case of small molecules, the stimuli-active component stands for therapeutics.

b Stimuli-activated therapeutics that induce direct biological effects after interaction of the material with the external stimuli.

c Drug release triggered by the stimuli, promoted *via* either structural changes in the (nano)carrier or cleavage of drug-carrier linkers. The active component here refers to the drug/API molecule exerting the therapeutic action.

d In this case, the drug molecule is not directly reacting to the stimuli but rather potentiates the therapeutic effect of the stimuli *via* sensitizing cells and tissues, sometimes even considered as a sensitizing agent.

**Fig. 5:**
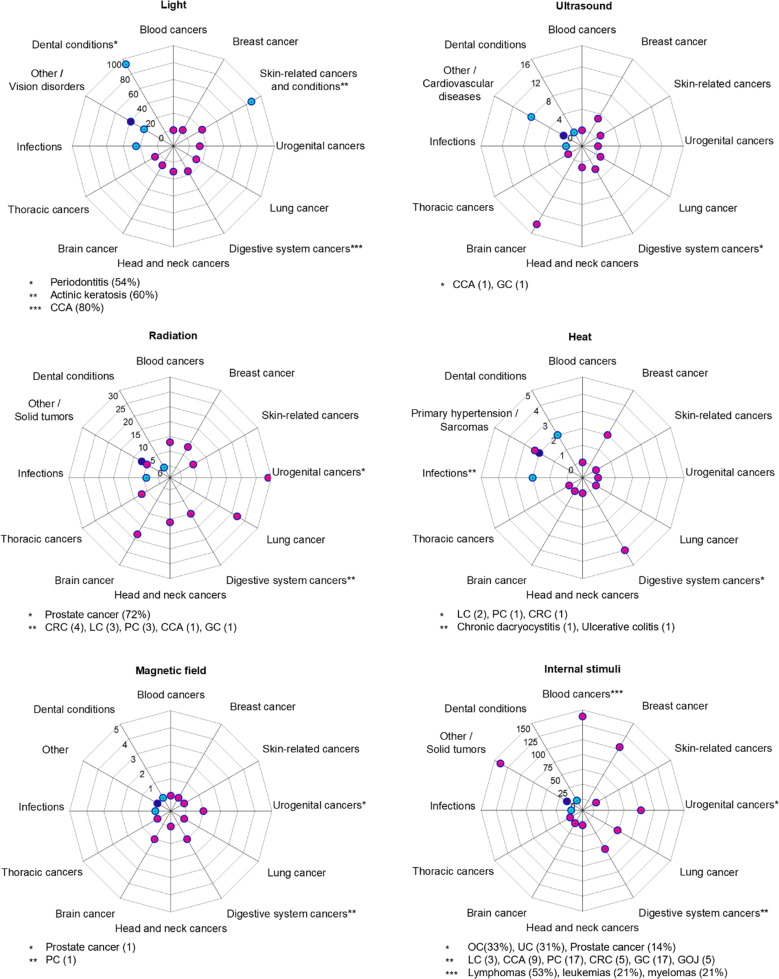
Distribution of the indications treated with stimuli-responsive materials in clinical therapy trials. The distribution of the most common conditions treated in clinical trials with stimuli-responsive materials during 10 years (2014–2024), categorized by the different stimuli (light, ultrasound, radiation, temperature, magnetic field, and internal stimuli). Data were obtained in October 2025, from the online database of clinical research studies, available at https://clinicaltrials.gov/. The data (1083 trials) were analyzed and manually sorted to exclude false positive cases, unrelated to stimuli-responsive therapeutics (*e.g.*, devices, or only diagnostic interventions). *Abbreviations*: CCA – cholangiocarcinoma (bile duct cancer), GC – gastric cancer, CRC – colorectal cancer, LC – liver cancer, PC – pancreatic cancer, OC – ovarian cancer, UC – urothelial cancer, GOJ – gastro-esophageal junction. Note: pink-colored points indicate cancerous diseases, cyan color represents non-cancerous diseases (specifically, vision disorders, dental and skin conditions, infections, cardiovascular diseases), and purple color represents other non-cancerous conditions.

Relying on the retrieved data (Fig. 4), we then examined the most common conditions treated with stimuli-responsive materials in clinical trials over the past decade, and cancer emerges as the most clinically assessed indication (Fig. 5).

Regarding the specific stimuli, light-based therapies are predominantly applied to superficial or accessible (surgical) sites due to the limited tissue penetration of light, with dental conditions, skin cancers and vision disorders among the most targeted indications. Phototherapies are also under investigation for treating bacterial, viral, and fungal infections. Interestingly, some deep-seated cancers, such as urogenital, bile duct and lung cancers, are also being addressed with light-based therapies, but often in tandem with surgical procedures using fiber-optic devices (*e.g.*, transperineal,^152^ bronchoscopic,^153^ or endoscopic^154^ techniques). Clinical trials with ultrasound-based therapies are focused mostly on brain cancers and cardiovascular diseases (Fig. 5). In this context, microbubbles are used to transiently open the blood–brain barrier for enhanced drug delivery, while sonoresponsive fluorescent dyes, including 5-ALA and porphyrins, are being evaluated for SDT. Radioenhancers are most explored for prostate and lung cancers, which are localized and amenable to intratumoral injections, and heat-responsive materials have shown promise in treating gastrointestinal cancers. Although the use of magnetic fields as a standalone therapeutic trigger is relatively recent, there is an upward trend in clinical trials in hard-to-treat malignancies, including brain, pancreas, and prostate cancers. Instead, materials responsive to internal stimuli are being deployed across a broad spectrum of solid and hematologic malignancies, with a special focus on breast, lung, urogenital, and gastrointestinal tumors.

**Fig. 6:**
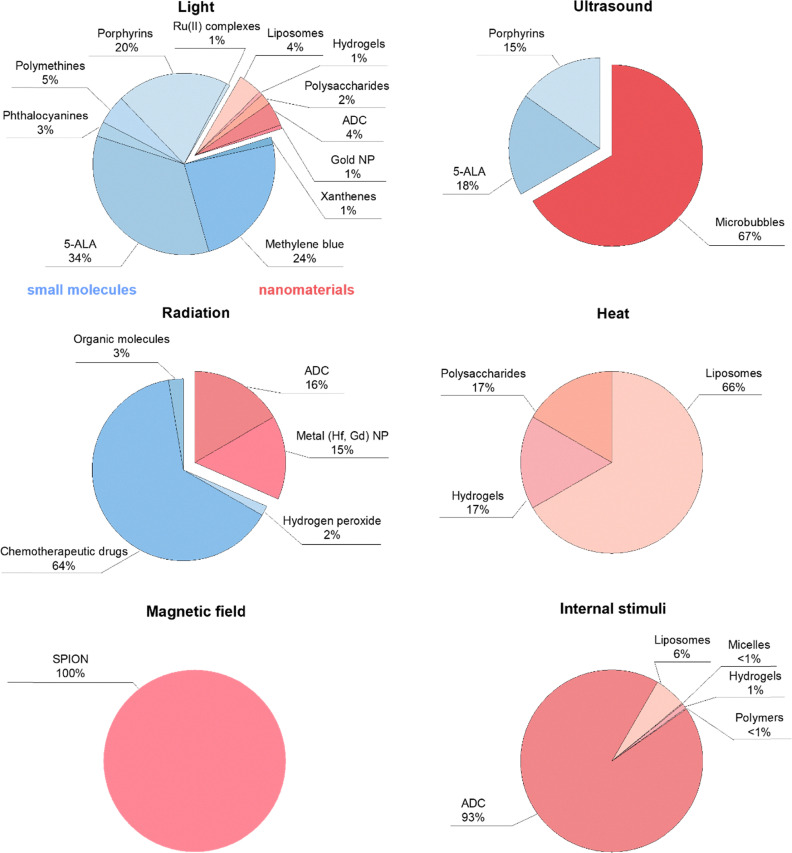
Landscape of stimuli-responsive material classes in clinical therapy trials. Distribution of the scope and type of materials responsive to light, ultrasound, radiation, heat, magnetic field, and internal stimuli (pH, enzymatic, redox) clinically evaluated for therapeutic purposes from 2014 until 2024. The blue color corresponds to small molecules, and the red color to nano- and microscale materials. Data were obtained in October 2025, from the online database of clinical research studies, available at https://clinicaltrials.gov/. The data (1083 trials) were analyzed and manually sorted out to exclude false positive cases, unrelated to stimuli-responsive therapeutics (*e.g.*, devices or only diagnostical interventions). *Abbreviations*: ADC – antibody–drug conjugates, NP – nanoparticles, 5-ALA – 5-aminolevulinic acid, SPION – superparamagnetic iron oxide nanoparticles. Note: the term “Organic molecules” (in the *Radiation* chart) refers to organic compounds that are not chemotherapeutics, such as anti-inflammatory agents.

To better evaluate the preclinical-to-clinical translation of stimuli-responsive therapeutics, we then specifically analyzed the distribution of the scope and type of materials explored for each stimulus in clinical trials (Fig. 6), as we did for the preclinical landscape (Fig. 3C). Overall, the relevance of nano- and micro-technology in clinically tested stimuli-responsive materials varies notably across stimulus type (Fig. 6 and Table 1). Their representation is relatively limited in light- (12%) and radiation-responsive (31%) clinical studies, where small-molecule therapeutics based on porphyrins, polymethines or 5-ALA still dominate the clinical trial landscape. In contrast, microbubbles dominate ultrasound-responsive therapies (67%), while clinical studies involving heat, magnetic fields, and internal stimuli essentially rely on nanotherapeutics.

### 3.1. Phototherapies

Phototherapies, including PDT, PTT, and PIT, have been evaluated in more than 360 clinical trials between 2014–2024, spanning applications in oncology, dermatology, and infectious diseases, assisting in the treatment of cancer and periodontal diseases, root canal disinfection, port-wine stain eradication and wound healing. As shown in Fig. 6 and Table 1, well-known photosensitizers, clinically approved over two decades ago, such as 5-ALA, porphyrins (Visudyne, a liposomal verteporfin formulation and the only clinically approved liposomal photosensitizer for age-related macular degeneration to date,^25^ Photofrin and Foscan), Indocyanine Green, and Methylene Blue, remain the focus of numerous trials (*e.g.*, NCT04964167 and NCT05359419). In fact, about 90% of current clinical studies still rely on these agents or analogs, owing to their proven clinical safety, efficacy and established regulatory profile (Fig. 6). For instance, Visudyne is being repurposed for oncological applications, including pancreatic cancer (*e.g.*, NCT03033225), prostate cancer (*e.g.*, NCT03067051, NCT06807359), breast cancer (*e.g.*, NCT02872064), and glioblastoma (*e.g.*, NCT04590664). Most of these trials are currently on the safety and dose escalation stage. Verteporfin (photosensitizer, and the active compound of Visudyne) has also attracted interest for light-independent applications as a YAP/TAZ pathway inhibitor.^155^ In terms of small molecule innovation, a few novel photosensitizers have entered clinical trials, including IR700 (a phthalocyanine derivative for NIR PIT of head and neck cancer) and Ruvidar (also known as TLD-1433, a ruthenium(ii) complex for PDT of non-muscle invasive bladder cancer, NCT03945162). Notably, intravesical 5-ALA (Metvix, CysView) is clinically approved for fluorescence-guided surgery and photodynamic diagnosis for bladder cancer.^156^ Despite the fact that there is a substantial number of preclinical studies incorporating photosensitizers in nanocarriers or using intrinsically activatable metal nanoparticles for light-based therapies (more than 18 000 publications in the last decade, Fig. 3C), their clinical translation has been limited. Gold nanoparticles^56^ are among the most frequently studied light-activated nanomedicines, yet they represent only about 1% in clinical trials of phototherapeutic materials (Fig. 6). Other metal-based systems, such as silver^157^ and copper nanostructures, integrated in a gel matrix with gold nanoparticles, have also reached early-phase trials for the treatment of indications like severe drug-resistant bacterial keratitis (NCT05268718, phase I).

#### 3.1.1. Clinical examples

*RM-1929* (also known as ASP-1929 or Akalux) is a tumor-specific (epidermal growth factor receptor (EGFR)-targeted) monoclonal antibody cetuximab conjugated to the silicon phthalocyanine dye IR700, activated by NIR light. Following intravenous administration, the ADC binds to tumor-specific antigens, and subsequent NIR light exposure induces photochemical reactions that cause rapid, irreversible cancer cell membrane damage.^29,72^ A phase I/IIa clinical trial of RM-1929 demonstrated the tolerability and potential efficacy in patients with recurrent head and neck squamous-cell carcinoma, with a confirmed overall response rate (ORR) of 26.7%,^158^ which promoted a followed-up phase III clinical trial (NCT03769506). In Japan, this ADC is clinically approved for the treatment of advanced and recurrent head and neck cancer under Akalux trademark.^159^ Additionally, a phase II clinical trial of RM-1929 (NCT05182866) has recently commenced in patients with newly diagnosed recurrent head and neck or cutaneous squamous cell carcinoma, combining PIT with fluorescence imaging. Overall, preliminary clinical data on RM-1929 show promising outcomes, with over two-thirds of patients in each cohort achieving disease control.^160^ Compared to the current standard-of-care for head and neck squamous-cell carcinoma (*i.e.*, platinum-based chemotherapy plus cetuximab, with an overall response rate of 55.9% and a complete response rate of 2.9%),^161^ RM-1929 demonstrates a higher overall response rate of 63.5% and an improved complete response rate of 17.3%.^160^

*Ruvidar*, a ruthenium(ii)-based PDT agent activated by a 532 nm-laser, is currently in phase II trials for non-muscle invasive bladder cancer that has not responded to Bacillus Calmette-Guérin therapy (NCT03945162), the standard first-line immunotherapy for this condition.^84^ Administered intravesically as a lyophilized suspension, Ruvidar has shown complete response rates of 54%, 38%, and 37% at 6, 12, and 15 months, respectively.^162^ For context, the clinically approved anti-PD1 therapy pembrolizumab achieves a 46% complete response rate at 12 months.^163^ While cross-trial comparisons should be interpreted with caution, these data suggest differences in response profiles over time between treatment modalities. If approved, Ruvidar would be the first intravesical, light-activated therapy for non-muscle invasive bladder cancer.^164^

*AuroShell*, a gold nanoparticle consisting of a 120 nm silica core coated with a thin layer of gold (∼15 nm), has been developed for PTT and extensively investigated in clinical trials, particularly for localized prostate cancer^56^ (Table 1). A phase I/II clinical trial using intravenously administered AuroShell prior to magnetic resonance or ultrasound imaging-guided laser irradiation demonstrated effective tumor ablation with minimal side effects (NCT04240639). The patients with localized prostate cancer experienced a complete tumor reduction confirmed by negative targeted biopsies with no abnormalities observed on the magnetic resonance imaging at the treated area,^30^ with follow-up at 6 months and 1 year, confirming durable response. These findings suggest that, if tested *vs.* standard-of-care and approved, AuroShell-based PTT may offer a viable focal treatment alternative to radical prostatectomy in selected patients.^30^ Beyond prostate cancer, AuroShell has also shown promise in preclinical models of lymphoma, where PTT enhanced immune responses by reducing T-cell exhaustion and improving synergy with immunotherapies.^165^

### 3.2. Ultrasound-mediated therapies

These therapies are gaining clinical relevance owing to their non-invasive nature, substantial penetration depth, and spatial precision.^85,87^ Notably, 50% of the clinical trial entries between 2014–2024 have been initiated only in the past three years, reflecting the accelerated interest in this therapy. Of the 33 trials registered, microbubbles account for 67% of the cases, followed by 5-ALA (18%) and porphyrins (15%), as shown in Fig. 6. Microbubble-mediated therapies build on decades of clinical use in cardiac imaging. Since the early 2000s, albumin-coated Optison and lipid-shelled SonoVue and Definity have entered clinical use.^166^ While the thicker protein shell of Optison may limit its oscillation behavior and, hence, sonopermeation efficiency, lipid-based formulations are generally considered more suitable for therapeutic applications owing to their greater acoustic responsiveness.^167^ Both agents are filled with fluorinated gases to reduce gas dissolution and prolong circulation time. However, they differ in shell composition: SonoVue primarily utilizes 16-carbon lipids, whereas Definity uses 18-carbon lipids, which confer greater mechanical stability and may support better stable cavitation without collapsing.^168,169^ Definity is more commonly employed in preclinical studies of blood–brain barrier opening (46 references *vs.* 20 for SonoVue, based on our search), which might be attributed to the narrower size distribution (1–3 µm *vs.* 1–10 µm). The narrower distribution, combined with greater mechanical stability, may reduce the risk of tissue damage during sonication, which is important in sensitive tissues like the brain.^170,171^ Microbubbles are increasingly investigated to enhance drug delivery across the blood–brain barrier.^172^ Numerous phase I and II trials have demonstrated their safety and feasibility, paving the way for ongoing phase III trials (NCT06496971 and NCT05317858). Other phase I trials are also investigating ultrasound-mediated radiosensitization in patients with malignant melanoma (NCT05620290), breast cancer (NCT04431674),^173^ and head and neck cancers (NCT04431648).^174^

Recent progress in brain-directed ultrasound platforms has sparked interest in SDT,^175,176^ and clinical trials are underway using systems such as ExAblate Neuro 4000 in glioblastoma and glioma patients (NCT04845919, NCT05362409, NCT04559685, NCT04469699), with one terminated due to funding challenges (NCT05370508) and one completed (NCT04845919).

#### 3.2.1. Clinical examples

*Definity* was used in combination with a skull-implantable device (SonoCloud-9) to transiently open the blood–brain barrier, followed by infusion of the albumin-bound paclitaxel nanoparticle Abraxane (nab-paclitaxel) in a recent phase I dose-escalation trial in recurrent glioblastoma (NCT04528680).^177^ After 68 treatment cycles, brain drug concentrations increased 3.7-fold for paclitaxel in sonicated *vs.* non-sonicated regions.^177^ Another phase I/II single-arm clinical trial exploited a similar approach, using carboplatin instead of Abraxane (NCT03744026). Thirty-three patients underwent 90 sonication cycles, and transient blood–brain barrier opening was achieved in 90% of the cases. Importantly, the results suggest that the therapeutic effect is stronger when carboplatin is injected prior to sonication compared to the carboplatin injected after sonication.^178^ Based on the outcomes of these two trials, its clinical efficacy is now being further assessed in a larger pivotal trial, which is expected to be completed in 2028 (NCT05902169).

*SonoVue* was investigated in the phase II trial NCT04146441 for the treatment of patients with inoperable pancreatic ductal adenocarcinoma receiving standard chemotherapy (FOLFIRINOX or nab-paclitaxel + gemcitabine). In the experimental arm, treatment was combined with a novel dual-frequency ultrasound transducer enabling simultaneous imaging (4.5 MHz) and therapy (0.35 MHz) at a mechanical index of 0.5.^179^ SonoVue agent was administered *via* nine intravenous boluses during a 35-minute ultrasound session. While safety was maintained, the median survival only increased modestly from 9.8 to 11.7 months in the sonopermeation-treated group. The authors attributed the limited therapeutic improvement to several factors, including variability in treatment timing, potentially overly destructive ultrasound parameters, and a low number of treatment cycles.^93,180,181^

*Sinoporphyrin sodium* has been recently evaluated in a phase II trial for SDT, targeting plaque inflammation in patients with symptomatic femoropopliteal peripheral artery disease (NCT03457662). After intravenous administration, a fraction of the sonosensitizer sinoporphyrin sodium accumulates in atherosclerotic plaques, where ultrasound activation triggers macrophage apoptosis and reduces inflammation.^182^ In a randomized, double-blind, sham-controlled study, localized ultrasound (1 MHz, up to 2.1 W cm^−2^) was applied to arterial plaques 4 hours after intravenous injection of sinoporphyrin sodium at 0.2 mg kg^−1^.^183^ Compared to sham treatment, SDT significantly reduced arterial inflammation (PET/CT: −0.72 *vs.* −0.19 target-to-background ratio; *P* < 0.001), improving walking functional performance (+118.8 seconds), increased ankle-brachial index, and lesion stenosis. These benefits persisted for up to 6 months, highlighting SDT as a promising non-invasive, macrophage-targeted therapy for cardiovascular lesions.

### 3.3. Radiation-mediated therapies

The analysis of 114 clinical trials conducted between 2014 and 2024 shows that chemotherapeutic drugs remain the most common adjuncts to radiation therapy (64%), followed by ADC (16%) involved in combinatorial regimens (Fig. 6). Metal nanoparticles, primarily based on hafnium and gadolinium, account for 15% of trials and have gained momentum mostly in the last decade. Since the discovery of X-rays in 1895, radiotherapy has been widely used as a monotherapy, leveraging its intrinsic ability to induce DNA damage and cytotoxicity. In recent years, however, chemoradiotherapy has emerged as a standard-of-care for several solid tumors, including head and neck, lung, esophageal, and rectal cancers.^184–187^ In glioblastoma, the combination of radiotherapy with temozolomide is commonly used post-surgery.^188,189^ These regimens capitalize on the radiosensitizing properties attributed to some chemotherapeutic agents such as cisplatin, carboplatin, fluorouracil, and gemcitabine, which, despite not being structurally responsive to radiation directly, have been reported to amplify DNA damage and enhance radiation therapy outcomes in combination treatment regimens.^9^ For instance, concurrent chemoradiotherapy (cisplatin and gemcitabine) combined with sintilimab improved event-free survival, with a higher rate observed in the sintilimab group compared to the standard therapy group (86% *vs.* 76%) in the treatment of locoregionally advanced nasopharyngeal carcinoma (CONTINUUM phase III trial; NCT03700476).^190^ In contrast, the KEYNOTE-412 phase III trial (NCT03040999)^191^ showed no event-free survival benefit from adding pembrolizumab to cisplatin-based chemoradiotherapy in head and neck squamous cell carcinoma, outlining the impact of the cancer type on the efficacy of such combined treatments. The clinical relevance of immune checkpoint inhibitors, particularly anti-PD1/L1 antibodies,^192^ also propelled combinational studies with chemoradiotherapy (NCT03700476, NCT03040999). Radiodynamic therapy is also being explored clinically, leveraging the favorable safety profile and prior clinical use of 5-ALA (Gliolan, orally administered porphyrin precursor), which acts as a radiosensitizer under ionizing radiation in glioblastoma (NCT05590689), though no results have been posted yet. Earlier trials (2000s) include motexafin gadolinium, a redox-active radiosensitizer, although its development was discontinued in 2007.^193^ Instead, radioenhancers like Hensify and AGuIX nanoparticles require an external radiation source to locally amplify cytotoxic effects at the tumor site, minimizing radiation exposure to healthy organs and reducing systemic and off-target toxicity.^2,3,56,59^

#### 3.3.1. Clinical examples

*Hensify* (also known as NBTXR3) are ∼50 nm-sized hafnium oxide nanoparticles EMA approved in 2019 for the treatment of locally advanced soft tissue sarcoma.^194^ The approval came after a phase II/III trial NCT02379845, which evaluated intratumorally administered Hensify activated by external beam radiotherapy compared to radiotherapy alone, as a preoperative treatment for locally advanced soft-tissue sarcoma. Complete responses were observed in 16% (14 of 87 patients) of patients in the Hensify group *versus* 8% (7 of 89 patients) with radiotherapy alone.^195^ The successful completion of phase III trials marked the first clinical validation of a radioenhancer providing therapeutic benefit in combination with standard radiotherapy. Following up on this, NCT04892173 phase III study was launched based on preliminary results (phase I) which reported to be safe with an objective response rate of 85.4% and complete response of 51.2% in elderly patients with locally advanced head and neck squamous cell carcinoma when treated with Hensify + radiotherapy. These findings supported the feasibility and tolerability of this combination in this patient group.^196^ Hafnium oxide nanoparticles are currently being evaluated in multiple clinical trials, either as a standalone treatment (head and neck cancer, metastatic liver and lung cancers) alongside radiotherapy (NCT04892173, NCT02721056, NCT04505267) or in combination with chemo- or immunotherapy (NCT04862455, NCT03589339). The potential of the intratumorally administered Hensify nanoformulation to treat metastases upon radiotherapy at distant, non-irradiated sites is mediated by the abscopal effect.^197^

*AGuIX*, a ∼5 nm-sized gadolinium-chelated polysiloxane-based nanoparticle, acts as both a radiosensitizer and contrast agent for magnetic resonance imaging.^59^ Administered intravenously, AGuIX accumulates in tumors and metastases and increases radiation-induced DNA damage. Its clinical evaluation has primarily focused on brain metastases, with a completed phase I trial (NCT02820454), where 13 of 14 evaluable patients showed tumor stabilization or reduction following treatment with radiotherapy in combination with AGuIX, with good tolerability and median overall survival of 5.5 months, although the absence of a control group precludes drawing any conclusions on the specific therapeutic benefit of AGuIX. Nonetheless, this combination was reported to be safe and feasible in patients with brain metastases.^198^ Ongoing phase II studies (NCT03818386, NCT04899908) aim to assess its efficacy in combination with whole-brain radiation therapy. Clinical results have demonstrated the accumulation of AGuIX nanoparticles in the brain and metastatic lesions.^198^ AGuIX is also being studied for proton therapy in a phase II trial for recurrent tumors (NCT04784221), however the study was terminated. Despite promising clinical developments, the company NH TherAguix (developer of AGuIX) entered judicial reorganization in 2025, raising uncertainties about the platform's future commercialization.^199^

*RiMO-301*, a hafnium-based nanoscale metal–organic framework also used as a radiosensitizer, entered clinical trials in 2023. It is intratumorally administered and, as Hensify, has been reported to be well-tolerated and to show preliminary activity in combination with radiotherapy. The study reported a 38.5% objective response rate (1 complete response and 4 partial responses of 13 patients) in a phase I trial for RiMO-301 in combination with palliative radiation for advanced tumors (NCT03444714).^200^ When considering additional combination with the PD1 inhibitor pembrolizumab, RiMO-301 objective response rate increased to 66.7% (4 of 6 patients), suggesting potential for combination strategies, although no final conclusions on efficacy can be drawn from a phase I study.

As a note, the two examples listed below cannot be technically considered as stimuli-responsive therapeutics, since they do not directly respond to or interact with the applied stimulus. Yet, they represent a widely established strategy, clinically evaluated in combinatorial settings, to potentiate radiotherapy in hypoxic cells and, thus, they are often considered as radiosensitizing agents.

*Dodecafluoropentane emulsion*, originally developed for oxygen therapy, was evaluated in a phase I trial (NCT02189109) for its potential to (re)oxygenate the typically hypoxic microenvironment of glioblastoma through systemic injection prior to radiotherapy. The treatment was well tolerated, and the median overall survival was 19.4 months with a 90% confidence limit, suggesting a potential survival advantage over the historical benchmark of 14.6 months with standard fractionated radiotherapy and temozolomide.^201^ However, due to the small cohort size (11 patients), further studies are necessary to determine the median progression-free survival.

*Hydrogen peroxide* renders a standalone approach to generate ROS and enhances the cytotoxic effects of radiation therapy for radioresistant tumors upon intratumoral injection in patients with locally advanced or recurrent breast cancer (NCT03946202), however this phase II study has not been completed yet. In a preliminary phase I trial, a subset of patients reported moderate pain after injection and skin toxicity comparable to RT alone when treated with KORTUC (0.5% of hydrogen peroxide in 0.83% sodium hyaluronate gel) + radiation, with 11 out of 12 demonstrating at least a partial or complete tumor response at a median follow-up of 12 months in this specific cohort.^202^ For disease stages 1 and 2, the administration of hydrogen peroxide (with the brand name KORTUC) achieved a 97.1% local control rate at 5 years, with minimal recurrence and metastasis.^203^ In contrast, outcomes in patients with disease stages 3 and 4 were less favorable (214 patients), with complete and partial response rates of 15% and 32%, respectively, and significantly lower median progression-free survival, underscoring reduced efficacy in advanced-stage disease.^204^

### 3.4. Magnetic field-mediated therapies

Clinical trials involving magnetic field-responsive therapeutics are mostly based on SPION. SPION were initially approved as magnetic resonance imaging contrast agents in 1996 for liver imaging (*e.g.*, Resovist). However, following the development of gadolinium-based agents capable of hepatobiliary phase imaging (MultiHance and Eovist), with superior pharmacokinetics compared to their colloidal iron oxide counterparts,^58^ most SPION formulations approved for imaging applications were discontinued.^205^ Despite their decline in imaging, SPION have been explored for other applications, particularly to treat iron-deficiency anemia, with ferumoxytol (Feraheme) obtaining FDA approval in 2009 for patients with chronic kidney disease,^58^ but also for sentinel lymph node mapping (*e.g.*, MagTrace).^205^ Moreover, SPION have also gained attention in the field of stimuli-responsive therapies (Fig. 6), particularly for magnetic hyperthermia.^12^ However, as of 2025, only 3 clinical trials involving SPION are listed in clinicaltrials.gov (Table 1).

#### 3.4.1. Clinical examples

*NanoTherm* is a SPION formulation administered intratumorally and activated by an alternating magnetic field, first approved by the EMA in 2010 as a medical device for the treatment of patients with recurrent glioblastoma multiform.^35^ In a clinical study,^34^ NanoTherm achieved median progression-free survival of 5.5 months, slightly lower than the 6.9 months reported for the Stupp protocol that exploits a combination of surgery, chemo- and radiotherapy,^206^ but superior to outcomes with the drugs lomustine and bevacizumab (3.4 months). The treatment was well tolerated, with minimal toxicity compared to standard chemotherapy. A similar study of NanoTherm in glioblastoma multiforme treatment was initiated in 2024 (NCT06271421) to evaluate side effects, overall survival, and progression free survival. However, no results have been posted to date. Separately, a phase IIB trial investigating NanoTherm for intermediate-risk prostate cancer treatment (NCT05010759, initiated in 2021) was terminated in 2023 due to inadequate enrolment and a shift in company priorities, with no public results available. Initial responses in the phase I trial, based on prostate-specific antigen levels, were limited in magnitude and duration,^207^ leading to the assumption that the intermediate results were moderate, thus making it hard to commercially compete with the standard-of-care prostate cancer treatment. Another phase I clinical trial posted in 2020 aimed to study the SPION with spinning magnetic fields in combination with neoadjuvant chemotherapy in osteosarcoma (NCT04316091), though no public updates have been released.

*NTT agent*, composed of γ-Fe_2_O_3_ nanoparticles, is currently being evaluated in a pilot study for the treatment of pancreatic ductal adenocarcinoma as part of the EU-based research project (NoCanTher).^208^ The NTT approach combines intratumoral SPION administration with magnetic hyperthermia to disrupt tumor stroma. The process also changes the physical characteristics of the tumor, making it more susceptible to anticancer drugs.^208^ Preclinical studies in patient-derived xenografts showed improved drug distribution and tumor shrinkage, leading to a first-in-human trial launched in 2022 at Vall d’Hebron Hospital (Barcelona, Spain).^209–211^ Using a custom-built magnetic field generator, the trial aims to assess safety, therapeutic synergy, tumor remodeling, and biomarker responses. Notably, as of 2025, the trial is not listed in a publicly accessible clinical trial registry such as clinicaltrials.gov.

### 3.5. Heat/thermotherapy

This treatment modality has been used for centuries, with modern studies dating back to the 1970s showing that heat can enhance the effects of radiotherapy and chemotherapy. In this section, however, the focus is specifically on materials that mediate heat-triggered drug release. Current clinical trials in this context (14 identified) show a strong preference for liposomes (66%; primarily, liposomal doxorubicin),^31^ followed by hydrogels (17%) and polysaccharides (17%) (Fig. 6). A key example is ThermoDox, a temperature-sensitive liposomal formulation of doxorubicin engineered to release the drug at temperatures ∼ 39–42 °C. Hydrogel nanoformulations embedding small-molecule therapeutics, such as mitomycin C, 5-fluorouracil, or nitazoxanide, exhibit sharper temperature-responsive release profiles. They typically rely on endogenous body temperature rather than externally applied hyperthermia to trigger drug delivery, which can facilitate eventual clinical implementation without the need for external devices that generate heat. Thermosensitive poloxamer-based gels loaded with 5-fluorouracil have also been developed to enhance drug adhesion and retention in colorectal cancer therapy.^212^ Administered *via* colonic transendoscopic enteral tubing, this system is designed to ensure uniform drug distribution across the tumor site. A phase II trial (NCT06385418) was initiated in 2024 to evaluate its potential to improve local drug retention and therapeutic efficacy.

#### 3.5.1. Clinical examples

*ThermoDox*, developed in the early 2000s, has been tested in combination with focused ultrasound and other localized heating methods for liver and breast cancer. However, subsequent clinical trials, including the HEAT trial initiated in 2008, revealed challenges in maintaining uniform intratumoral temperatures, due to heterogeneity in tumor vascularization and technical limitations in sustaining localized heat. The clinical investigation of ThermoDox continued with the OPTIMA phase III trial (NCT02112656), which was initiated in 2014 for the treatment of hepatocellular carcinoma in combination with radiofrequency ablation. However, results published in 2024 showed no significant improvement in progression-free survival, with a median value of 19.3 months in the ThermoDox group *versus* 16.8 months in the control group receiving placebo infusions. Inconsistent heating resulted in inefficient drug release, reducing its therapeutic efficacy, which, combined with suboptimal clinical trial designs, hindered positive clinical outcomes.^213^ Additionally, doxorubicin was observed to leak under non-heating conditions, leading to off-target accumulation.^214^ That issue promoted the development of THE001, another temperature-triggered doxorubicin-loaded liposome formulation designed to be more stable than ThermoDox. THE001 contains the 1,2-dipalmitoyl-*sn*-glycero-3-phosphodiglycerol (DPPG) lipid instead of the lysolipid 1-stearoyl-2-hydroxy-*sn*-glycero-3-phosphatidylcholine (MSPC) used in ThermoDox. THE001 releases its cargo at temperatures exceeding 40 °C and showed improved stability in human serum in a head-to-head comparison with a ThermoDox-mimicking formulation.^214^ In 2023, a phase I dose-escalation study of THE001 combined with regional hyperthermia for the treatment of advanced or metastatic soft tissue sarcoma was initiated (NCT05858710).

*UGN-10X* is a series (*X* = 1–4) of thermoresponsive hydrogels containing mitomycin C, developed for intravesical bladder cancer treatment. Based on a proprietary reverse-thermal hydrogel (RTGel) technology,^215^ these formulations remain liquid at room temperature and gel upon exposure to body temperature, conforming to patient-specific pelvicalyceal and ureteric anatomy.^216^ UGN-103 (NCT06331299) is currently in phase III clinical trials for low-grade, intermediate-risk non-muscle invasive bladder cancer, designed to enhance local drug exposure while minimizing systemic toxicity. The trial builds on promising results from its predecessor, UGN-102 (NCT05243550), which showed a complete response rate of 79.2% at 3-month follow-up,^217^ surpassing the 64% rate typically achieved with standard transurethral resection of bladder tumors.^218^ Most observed adverse events were mild or moderate. If validated, UGN-103 may offer a non-surgical therapeutic alternative, reducing reliance on transurethral resection.

### 3.6. Internal stimuli-triggered therapies

Currently, more than 550 clinical trials involve pharmaceuticals triggered by internal stimuli, with a growing trend reflecting sustained interest and investment. Although more than 40 000 (nano)materials of different classes (*e.g.*, antibodies, liposomes, hydrogels, carbohydrates) have been explored for internal stimuli-triggered drug delivery between 2014 and 2024 (Fig. 3C), clinical trials are currently heavily dominated by ADC (93%) (Fig. 6). ADC translation and clinical impact is already well covered in dedicated reviews, to which the reader is also referred.^16,19,139^ However, it is worth mentioning that there are several major directions propelling ADC in clinical trials: (i) ADC having novel target antigens (NCT02631876, NCT04154956),^16^ (ii) ADC having dual payloads (NCT06963281),^140^ and (iii) ADC combination regimens with immune checkpoint inhibitors (NCT01896999, NCT02131064, NCT04042701),^219,220^ chemotherapy (NCT04024462, NCT05456685),^221^ and radiotherapy (NCT06210490, NCT05115500).^221^ Also, two new approaches are gaining traction: probody-drug conjugates^139^ (NCT03149549, NCT03543813, NCT04681131) and bispecific ADC, which have reached first-in-human clinical trials (NCT05194982).^139,222^

Despite extensive preclinical research on internal stimuli-responsive systems (Fig. 3C),^7,44^ only one micellar formulation (NC-6300) and no liposomal formulation explicitly designed for internal stimuli activation have entered clinical trials in the past decade. Notably, 6% of related clinical trial entries involve liposomal doxorubicin (Fig. 5), which, despite being sensitive to pH changes that promote drug release, is not categorized as a stimuli-responsive system.^223^ It should be acknowledged that such mechanistic details may not always be relevant to be reported by the inventors, or they fall under intellectual property protection and are not always disclosed in clinical trial databases or associated publications.

#### 3.6.1. Clinical examples

*Mirvetuximab soravtansine* (MIRV) is an ADC composed of an antifolate receptor α (FRα) monoclonal antibody, a redox-cleavable disulfide linker (sulfo-SPDB), and the maytansinoid DM4 drug, a potent tubulin-targeting antimitotic agent, with a drug-to-antibody ratio of 3.5.^224,225^ The single-arm phase II SORAYA trial (NCT04296890),^225^ which evaluated Mirvetuximab soravtansine monotherapy in patients with high-grade platinum-resistant epithelial ovarian cancer, primary peritoneal, or fallopian tube cancer, already led to fast-track FDA approval of MIRV in 2022.^224^ A follow-up phase III MIRASOL trial (NCT04209855) was designed to compare this ADC with standard-of-care chemotherapy (paclitaxel, pegylated liposomal doxorubicin, or topotecan), and MIRV demonstrated improvements in an objective response rate (36.1% *vs.* 14.6%), median progression-free survival (5.9 *vs.* 4.3 months) and overall survival (16.5 *vs.* 12.8 months).^226^ The results led to FDA approval for treatment in 2024.^227^

*BL-B01D1*, a first-in-class epidermal growth factor receptor (EGFR)–HER3 bispecific antibody–drug conjugate, is composed of a bispecific antibody (an anti-EGFR human IgG1 antibody fused to two anti-HER3 human single-chain fragment variables *via* the glycine-serine linker), a cathepsin-cleavable tetrapeptide-based linker, and the toxin Ed-04 (a camptothecin derivative, topoisomerase I inhibitor), with a high drug-to-antibody ratio of 8. The ongoing phase I trial (NCT05194982) is evaluating BL-B01D1 monotherapy in patients with locally advanced or metastatic solid tumors. Preliminary results from 195 patients indicate an acceptable safety profile (though moderate-to-high side effects were reported).^222^ Treatment responses vary depending on cancer type (objective response rate equals 52.5% for patients with EGFR-mutant NSCLC, 31% for patients with EGFR wild-type NSCLC, and 44% for patients with nasopharyngeal carcinoma). The observed safety profile supports further clinical evaluation of BL-B01D1.^222^

*CX-2029* is a probody-drug conjugate composed of a masked anti-CD71 monoclonal antibody, an enzyme (protease)-cleavable valine–citrulline (Val–Cit) linker, and the cytotoxic payload monomethyl auristatin E (MMAE), with a drug-to-antibody ratio of 2.^228^ The antibody is sterically shielded by a peptide mask, which is cleaved in the tumor microenvironment by proteases, enabling local activation while minimizing systemic toxicity. In the first-in-human phase I/II trial PROCLAIM (NCT03543813), CX-2029 was administered to patients with advanced solid tumors. At the recommended phase II dose (3 mg kg^−1^), partial responses were observed in 4 of 13 patients with NSCLC and in 3 of 11 patients with head and neck squamous cell carcinoma.^228^ These outcomes appear encouraging to those of conventional chemotherapy in relapsed/refractory NSCLC and HNSCC, where objective response rates typically remain below 10–15%. The trial highlighted the feasibility of targeting CD71, a previously undruggable antigen due to its ubiquitous expression, through conditional activation in the tumor microenvironment. To date, however, no probody-drug conjugate has progressed beyond early-phase trials, and no phase III studies have been initiated.

*NC-6300* (NCT03168061) is a pH-responsive micellar formulation comprising the chemotherapeutic agent epirubicin conjugated to a poly-aspartic acid backbone *via* an acid-labile hydrazone bond, enabling selective drug release in acidic tumor environments. Developed for soft tissue sarcoma (59%) and other solid tumors, NC-6300 showed a modest improvement in median progression-free survival of 5.4 months^229^ compared to 4.6 months for doxorubicin.^230^

*CA102N* is a polymer conjugate of hyaluronic acid and nimesulide derivative, bound *via* a lysosomal degradable linker for targeted delivery to CD44-overexpressing tumors.^231^ The conjugate remains stable in the bloodstream and releases nimesulide intracellularly following lysosomal degradation, enabling COX-2 inhibition within tumor cells.^232^ In a phase I trial,^233^ CA102N showed no dose-limiting toxicities, with Grade ≥3 treatment-emergent adverse events occurring in only 8% of patients and a median progression-free survival of 3.7 months. A phase II trial (NCT06039202) is currently evaluating its safety, tolerability, and efficacy in combination with trifluridine/tipiracil in colorectal cancer. However, the observed median progression-free survival remains significantly lower than that of standard first-line therapies, such as FOLFOX/FOLFIRI (7–11 months), underscoring the need for further optimization.

## 4. Challenges and solutions

Despite extensive preclinical research, only ∼ 1% of stimuli-responsive therapeutics progress to clinical trials, reflecting a significant translational gap in the field. In line with the translational journey of many other pharmaceuticals, several pathophysiological, pharmaceutical, clinical, and regulatory barriers must be overcome to bridge the bench-to-bedside gap for stimuli-responsive therapeutics. One of the first challenges lies in the scope of early-stage research in materials science, often focusing on optimizing material properties *(e.g.*, structural and general physicochemical properties such as spectral tuning, ROS generation capabilities, or stability of sensitizing agents) over identifying concrete medical needs and considering clinical feasibility aspects. A material-focused approach may come with the risk of overlooking critical factors in drug development, such as pharmacokinetics, scale-up manufacturing feasibility, cost efficiency, or end-user acceptance. Many of these aspects become even more challenging when involving nano- or microtechnology in the design of stimuli-responsive therapeutics.^234^

Besides standard pharmaceutical and translational challenges, stimuli-responsive therapeutics face additional, specific hurdles, which also depend on the stimulus being exploited (Table 2). For instance, light-based therapies are clinically limited by the shallow tissue penetration and the need for complex laser devices, or ultrasound-responsive materials must be tailored to respond to clinically relevant frequencies. Radiation- and magnetic field-sensitive systems require precise control over the (nano)particle properties and stability. Thermal-triggered platforms face challenges in achieving consistent and localized temperature elevation *in vivo*, and internally activated therapeutics must contend with the heterogeneous distribution of stimuli (*e.g.*, enzyme levels) across different tissues and organs, and between patients. Besides these abovementioned stimuli-specific limitations, the need for specialized (and often costly) equipment to generate the stimuli, device setup optimization, and optimal integration into existing treatment protocols adds up to translational challenges, complicates clinical adoption, and limits their value proposition for stakeholders and market interest.

**Table 2:** Challenges and opportunities in the development and clinical translation of stimuli-responsive therapeutics

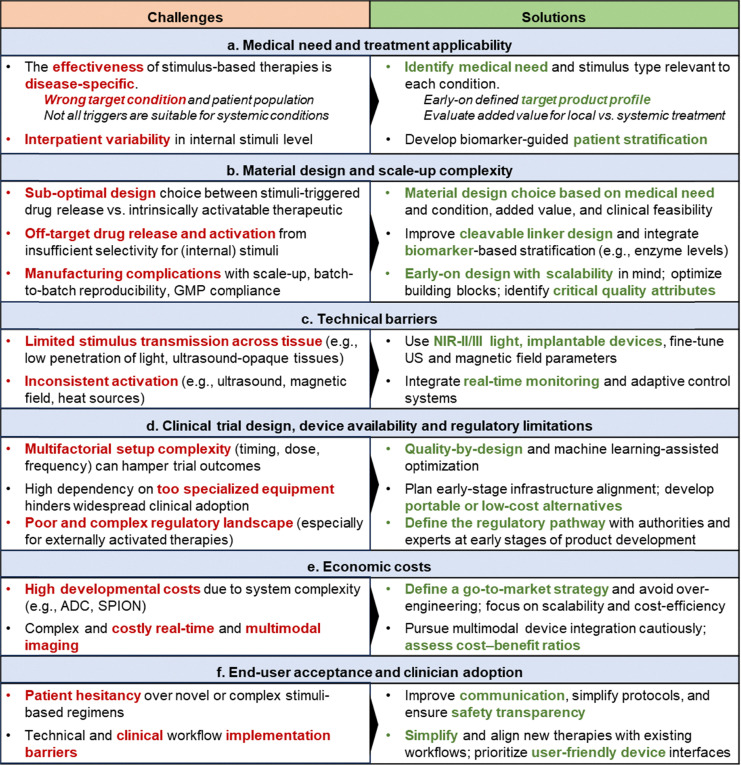

To improve the translational success rate in the future, materials research and development should be increasingly guided by clearly defined medical needs and a better understanding of the currently available therapeutic landscape. Together, this will enable a more realistic path toward clinical application.

### 4.1. Medical need and treatment applicability

Stimuli-responsive therapeutics hold high potential to spatiotemporally control the therapeutic action while minimizing off-target effects. Yet, their treatment performance and applicability are disease- and stimulus-specific. A trigger that is effective for treating one medical condition may be ineffective or impractical in another. Hence, identifying the concrete medical needs to address and the pathophysiological features of the specific indication can help to guide the design of novel stimuli-responsive therapeutics and maximize their eventual clinical relevance.

#### 4.1.1. Local *vs.* systemic therapies

In general, external stimuli-responsive therapies show higher potential for localized treatments, where stimuli can be precisely applied to specific body regions and maximally co-localized with the therapeutic/sensitizing agent. This makes them particularly suited for indications such as solid tumors, local infections, ocular diseases and dermatological disorders. In those settings, stimuli-responsive therapeutics can be locally administered (*e.g.*, intratumorally, intraocularly or intradermally), and the corresponding stimuli controllably and locally applied on the disease region, maximizing therapeutic responses. However, their utility in systemic diseases, such as metastatic cancers, autoimmune conditions, or hematological malignancies, remains limited due to challenges in controllably delivering the corresponding stimulus throughout the body without causing off-target effects. Although some external triggers, such as localized radiation, have demonstrated to exert systemic immunomodulatory effects (*e.g.*, *via* the abscopal effect),^235^ these responses are highly variable between individuals and not yet consistently reproducible in clinical settings.

In contrast, internal stimuli-responsive materials, activated by intrinsic biochemical cues such as pH, enzymes or redox gradients, are more amenable to systemic administration and therapies, as they can potentially be engineered so that the activation occurs selectively within the diseased tissues. Yet, the heterogeneity of the endogenous triggers between tissues (both diseased and healthy) and individuals poses a major translational barrier to ensure consistently effective and reproducible responses across patients. Variability in enzyme expression, pH, or redox states can compromise both selectivity and efficacy. A promising strategy to enhance the therapeutic efficacy of internal stimuli-responsive drugs and nanodrugs relies on the identification of biomarkers that correlate with disease states, including acidity, enzyme levels and redox state, and that eventually allows to predict and maximize patient responses to such therapies. Exploring tissue biomarkers and personalized medicine approaches, including the use of companion diagnostics, could help determine patient-specific biochemical features, allowing for tailored treatment strategies.^22^ Companion diagnostics, relying on imaging and enzyme level assays, may serve as gatekeepers for pre-treatment patient selection, helping identify those most likely to benefit from specific stimuli-responsive treatments.^236^

### 4.2. Material design and scale-up complexity

Once the need for stimuli-responsive therapy has been identified, the next step is to design and engineer the corresponding material, which also comes with specific challenges.

#### 4.2.1. Stimuli-triggered delivery of drug payloads *vs.* stimuli-activatable nano-/micromaterials

A key design consideration concerning stimuli-responsive systems is whether (a) to develop a carrier material that delivers a cytotoxic payload after the application of a stimulus (*e.g.*, *via* cleavage of a linker, such as in ADC),^16,19,66^ or (b) to structurally engineer a (nano-)material that displays intrinsic bioactivity after the application of a stimulus (*e.g.*, photosensitizers,^1^ radioenhancers,^2,3^ and magnetothermal agents).^11^ Each approach presents its own challenges, which need to be considered before designing the stimuli-responsive therapeutic entity.

On the one hand, stimuli-triggered cytotoxic drug delivery aims to employ carrier systems to spatiotemporally control drug delivery, enhancing target-site accumulation and effects, while minimizing off-target damage. This can be exploited for both external (*e.g.*, ThermoDox) and internal stimuli (*e.g.*, ADC), although it has been more clinically advanced for the latter. Employing biocompatible, clinically relevant delivery systems with approved cytotoxic drugs can streamline pharmaceutical development and regulatory trajectories.^234^ However, because internal stimuli are not always exclusive to diseased tissues, partial off-target release of cytotoxic drugs is often observed, limiting the therapeutic outcomes. Optimization of carrier systems, drugs, and stimuli-sensitive linkers will yield incremental improvements, albeit complete elimination of off-target toxicity using internal stimuli remains challenging. In this regard, exploration of alternative stimuli-sensitive linkers (*e.g.*, carbamate or aminoacrylate for light cleavage in photoactivated chemotherapy,^237^ or oxanorbornene derivatives for ultrasonic cleavage)^238^ allows to develop novel materials and open therapeutic opportunities, albeit some of these may not yet align with clinically relevant wavelengths or frequencies.

Combining different approaches,^9,239–241^ such as ultrasound-assisted drug delivery, chemoradiotherapy or immunotherapy, is also under clinical investigation, aiming to enhance local drug concentration at the target site and potentially reduce off-target toxicity. However, whether these strategies allow for lower, and thereby safer, drug doses remain context-dependent and require further clinical validation. A personalized approach targeting tumor-specific neoantigens offers another promising pathway, with early data suggesting high selectivity and comparable costs to conventional therapies.^242,243^

In contrast, directly activatable (nano-)materials, as photosensitizers, sonosensitizers, and radioenhancers, are designed to remain biologically inert until the application of an external stimulus, thus offering greater control over the therapeutic action. However, as discussed above, their efficacy is tied to optimal *in vivo* co-localization of the material with the applied stimulus. Insufficient stimulus application time or intensity (whether light, ultrasound, or magnetic field) can lead to suboptimal therapeutic outcomes, and once the stimulus ceases, its effect often terminates, thus requiring repeated or prolonged stimulation. In addition to these constraints, the engineering complexity of the materials to ensure adequate responses in human tissues can seriously compromise formulation reproducibility, stability and biocompatibility.

Therefore, balancing the advantages and disadvantages of each therapeutic material design strategy crucially requires the consideration of the intended targeted medical indication and the associated pharmaceutical developmental trajectory. Importantly, the choice of the material platform itself already influences the translational potential of the final stimuli-responsive formulation. Therapeutic systems based on approved, clinically tested, and well-characterized nano- and microscale platforms such as liposomes, lipid nanoparticles, certain polymers, or antibodies, generally benefit from more predictable pharmacokinetics and established safety profiles, as well as a clearer regulatory pathway, together facilitating clinical development and translation. In contrast, newly developed or less clinically validated materials (*e.g.*, certain metal–organic frameworks or complex hybrid nanostructures) may face additional challenges related to unknown biocompatibility, long-term safety, large-scale manufacturing, and regulatory acceptance. While some of these emerging systems may offer unique functional advantages, their increased design complexity can delay translation and clinical impact. Hence, selecting a material platform that balances functional performance with translational feasibility can help address the targeted medical need while accelerating clinical implementation.

#### 4.2.2. Design complexity *vs.* scalability, reproducibility and manufacturing costs

Scaling up stimuli-responsive materials from lab-scale synthesis to clinical-grade production introduces additional translational challenges, particularly for nano- and microscale therapeutics.^234^ Ensuring batch-to-batch reproducibility and control over the critical quality attributes during scale-up manufacturing, including on the stimuli-responsiveness effectiveness, is critical for pharmaceutical development; alongside considerations of (long-term) storage stability and transport logistics.^244,245^ These factors are closely monitored under GMP guidelines, where not only the final formulation, but also each “building block” (*i.e.*, polymers, ligands, and active agents) must meet stringent quality standards.^246^

### 4.3. Technical barriers

When the therapeutic agent is designed, it should maintain its efficacy not only in *in vitro* setups, but also in *in vivo* (clinical) settings. The core technical challenge lies in the limited and variable transmission of external stimuli through biological tissues, which hampers the precise activation of stimuli-responsive therapies at the intended site of action.

#### 4.3.1. Interaction of the stimulus with biological components

Stimuli interact with biological components when applied, which can block or reduce the intensity of the stimuli and thereby limit the effectiveness of the therapy.^76^ Light-based therapies exemplify this limitation. Despite their long developmental history, they are still confined to superficial applications (*e.g.*, treatment of skin diseases or head and neck cancer) or intraoperative settings, due to their poor tissue penetration capability. Although fiber optic tools such as bronchoscopes and endoscopes can deliver light deeper into the body, this compromises the minimally invasive nature of light-based therapies. Recent advances, including implantable micro-optical devices (*e.g.* µLED)^247,248^ co-administered with photosensitizers, could offer a promising route to deeper and more precise light delivery. Additionally, novel NIR-II/NIR-III light-activatable materials that can reach deeper tissue penetration could open opportunities for treating deep-seated malignancies in the coming years. Ultrasound-based therapies, which overcome the tissue-depth limitations of light, require instead precise tuning of frequency and intensity to balance therapeutic efficacy with safety. Inconsistent ultrasound parameters can lead to insufficient drug release or unintended tissue damage, necessitating real-time monitoring and advanced imaging guidance. Also, some tissues are opaque to US application, *e.g.*, the air-filled lungs, preventing ultrasound signal propagation.^249^ Magnetic field-based therapies, such as magnetic hyperthermia, face technical constraints in generating uniform magnetic fields across large tissue volumes, making precise and safe heat induction difficult to achieve.^250^

### 4.4. Clinical trial design, device availability and regulatory limitations

Following the preclinical development of the new therapeutic agent, this must be assessed clinically, where planning the optimal clinical trial design is crucial to achieve successful outcomes.

#### 4.4.1. Clinical trial design and optimization

Clinical trials require dose escalation studies to establish acute and long-term toxicity, dose-limiting toxicity, the maximum tolerated dose, and to outperform standard-of-care regimens.^251^ Stimuli-responsive therapeutics add another layer of complexity here, requiring optimal setup regarding activation parameters in clinical settings (including stimuli dose, time, intensity, and frequency) to ensure optimal therapeutic efficacy and safety. This can compromise clinical trial outcomes and hinder approval, as exemplified by the temperature-sensitive ThermoDox formulation, where flawed clinical trial designs, mostly on suboptimal hyperthermia settings, led to mixed outcomes.^22^ Standardized activation protocols are thus essential to ensure reproducibility, yet complete optimization is rarely achievable even at later clinical trial stages. Given the multifactorial nature of these therapies, more structured optimization protocols, including Quality-by-Design and Design-of-Experiments principles,^252^ should be further exploited, at least for preclinical stages.^253^ This may allow to better identify optimal stimuli application parameters, which, together with the implementation of computational methods and machine learning strategies,^254,255^ can facilitate the transfer of the application parameters into clinical settings.

#### 4.4.2. Device availability

Another key limitation for external stimuli-responsive therapeutics is the dependency on specialized equipment, such as laser systems, X-ray or magnetic field generators, which may not be readily available across healthcare facilities, particularly in peripheral hospitals. This infrastructure barrier can hinder widespread clinical adoption of these types of therapies. Moreover, successful integration requires further harmonized training protocols and workflow-compatible device interfaces to avoid disrupting standard clinical operations. As such, early-stage planning should also account for medical needs and infrastructure constraints to ensure clinical feasibility.

#### 4.4.3. Unclear regulatory pathways

Healthcare products can be classified into different categories, such as medicinal products, medical devices, or combination products, which integrate both components. However, current regulatory frameworks require investors to choose either the medical device or medicinal product approval pathway, each with distinct requirements: devices typically demand proof of mechanical safety, performance, and risk classification, while medicinal products require extensive clinical efficacy, pharmacokinetics, and toxicity data. For stimuli-responsive systems, especially those relying on external activation (*e.g.*, light, ultrasound), it is often unclear which regulatory route applies, as these technologies inherently combine physical activation with therapeutic effect.^256^ The most effective strategy is to engage early on with regulatory authorities to clarify the primary mode of action and define the most appropriate regulatory path, minimizing delays and streamlining product development.

### 4.5. Economic costs

A major challenge is that both the high manufacturing and infrastructure costs of stimuli-responsive therapies, especially complex nanomaterials and device-dependent modalities, can make it difficult to attract investors and reach widespread clinical adoption.

#### 4.5.1. Manufacturing costs

While systematic data on nanomedicine manufacturing expenses remain scarce, ADC, comprising just three core components (antibody, linker, drug), offer a relevant benchmark. Even these relatively simple constructs present affordability challenges for lower- and middle-income classes.^257^ Thus, the development of over-engineered systems can become economically not viable from a manufacturing and clinical implementation perspective,^258–260^ together resulting in difficulties to raise capital investment, critically limiting their translation.

#### 4.5.2. Device costs

While LED-based light sources and ultrasound devices are relatively inexpensive (USD 1000–10 000, and USD 100 000–300 000, respectively), other devices, such as magnetic resonance imaging-guided field generators and medical linear accelerators, are significantly costly (both can exceed USD 1 million, with additional maintenance costs). This obviously creates an additional financial barrier for healthcare facilities. The magnetic-activatable SPION formulation NanoTherm is a relevant example collecting numerous translational barriers, since, despite its EMA approval as a medical device in 2010,^35^ it has never advanced to phase III trials, with limited clinical data. Key barriers include the need for specialized infrastructure and equipment for precise intratumoral SPION injection into brain tumors, dedicated alternating magnetic field generators for hyperthermia activation, and trained personnel to administer the therapy. Therefore, only a small number of hospitals and research centers are able to offer NanoTherm therapy, which, in addition, limits the number of treated patients, and consequently, the volume of clinical data available from the trial.

#### 4.5.3. Technological integration

Many external stimuli-responsive therapies also require real-time imaging guidance to ensure accurate targeting of therapeutics, necessitating the integration of multimodal platforms, such as ultrasound combined with magnetic resonance imaging or laser-endoscope systems.^261^ While such combinations can significantly enhance treatment monitoring and precision, they also add technical and financial complexity, effectively increasing infrastructure and maintenance costs.^262^

Future research should therefore prioritize the development of simpler and more cost-effective alternatives, including portable ultrasound devices and low-cost magnetic field generators, to make stimuli-responsive therapies more financially viable for routine clinical use. For broad clinical adoption, comprehensive cost-benefit analysis will be essential to demonstrate that the clinical advantages of stimuli-responsive therapies justify the added economic burden when compared to more conventional treatments.

### 4.6. End-user acceptance and clinician adoption

The success of stimuli-responsive therapies depends not only on the scientific and technical advances, but also on ensuring patient acceptance and broad clinical adoption.

#### 4.6.1. Patient concerns

External stimuli such as radiation or magnetic fields can raise safety and tolerability concerns in patients, potentially impacting adherence.^263^ Multistep treatment regimens, such as systemic drug administration followed by localized stimulus, may be perceived as burdensome or risky. Ethical concerns also emerge when these therapies resemble conventional modalities like chemotherapy or radiotherapy, which patients may associate with toxicity or long-term harm.

#### 4.6.2. Clinician adoption

Adoption in clinical settings depends on the ease of use and compatibility with existing workflows. Healthcare professionals must be trained to use specialized activation devices and interpret real-time response monitoring information. A therapy that is too technically demanding may struggle to gain traction in clinical settings. In this regard, internal stimuli therapies administered through infusions may appear more easily implementable and beneficial, resulting in faster and wider adoption by clinicians. On the contrary, intratumoral injections (*e.g.*, for NanoTherm, Hensify, and RiMO-301) are not always straightforward depending on the localization of the treatment site, and require specially trained personnel, hence compromising their regular clinical use unless they demonstrate clear superiority over existing therapies.

Overall, improving end-user compliance requires more effective science communication, increasing the general knowledge for both patients and clinicians, transparent dissemination of safety and efficacy data, and the development of streamlined, user-friendly treatment protocols that minimize the burden on both patients and healthcare providers.

## 5. Outlook

Stimuli-responsive therapeutics have already achieved considerable clinical impact, with several products used in the treatment of cancer, dental conditions, vision disorders, and infectious diseases. While small-molecule fluorescent dyes remain relevant, the advances in nano- and microtechnology have driven the clinical adoption of novel materials such as SPION, ADC, Hensify nanoparticles, and SonoVue or Definity microbubbles. These materials paved the way for new therapeutic modalities, including sonopermeation, radiotherapy-enhancement, magnetically induced hyperthermia, and improved drug delivery and targeting control. A comparative summary of major stimuli, including advantages, limitations, trade-offs, and translational status, is provided to contextualize the clinical translation landscape of different stimuli and stimuli-responsive nanomedicines and microscale therapeutics (Table 3).

**Table 3:** Comparative summary of stimulus modalities, trade-offs, and translational status in stimuli-responsive nanomedicines and microscale therapeutics

Stimuli	Advantages	Challenges	Main trade-offs	Common indications	Translational status/potential	Clinically relevant examples
Light	Non-invasive, cost-effective, high spatiotemporal control, simple implementation	Limited tissue penetration, oxygen-dependent	High precision *vs.* low penetration depth	Superficial cancers, dental and skin conditions, vision disorders, intraoperative	Clinically established/High ✓✓✓	5-ALA, porphyrins (*e.g.*, liposomal Visudyne), methylene blue (approved)
Ultrasound	Non-invasive, deep tissue penetration, high spatiotemporal control, potential for blood–brain barrier opening	Understanding and fine-tuning of ultrasound parameters to maximize response; artifacts and low/no penetration through lungs, bone, and gas filled bowel	High penetration *vs.* parameter optimization	Brain diseases, Tumor vasculature perfusion	Clinical trials/High ✓✓✓	Microbubbles (approved for imaging and in trials for therapy)
Radiation	Non-invasive, deep tissue penetration, clinically well established	Mostly metal-based materials, administration routes	Physical dose amplification *vs.* delivery and distribution limitations	Urogenital, lung, and brain cancers	Clinically established/Medium ✓✓	Hafnium oxide nanoparticles (EMA approved)
Heat	Minimally invasive, cost-effective	Poor spatiotemporal and temperature control, side effects	Simplicity *vs.* complex stimulus control	Liver and breast cancers	Clinical trials/Medium ✓✓	ThermoDox, UGN-103 hydrogel (both in trials)
Magnetic field	Non-invasive, deep tissue penetration	Metal-based materials, side effects, difficulty to locally apply in deep tissues	High penetration *vs.* high device and running costs	Brain, prostate, pancreatic cancers	Clinically established but limited usage/Medium ✓✓	SPION (*e.g.*, NanoTherm; EMA approved)
Internal stimuli	Versatile, modular design, simple implementation, no external device required	Off-target activation and side effects, limited specificity for heterogenous tumors	Efficacy *vs.* toxicity	Liquid and solid cancers	Clinically established/High ✓✓✓	Antibody–drug conjugates (>10 approved), prodrugs

The successful translation and implementation of stimuli-responsive therapeutics depend on strong cross-disciplinary collaboration to align therapeutic material design with concrete medical needs, infrastructure availability, and patient expectations, while ensuring that the conceptualization of novel stimuli-responsive therapeutics, especially for more complex nano- or microscale materials, is driven by the medical needs, disease features and clinical implementation challenges, striving for material simplicity while ensuring adequate therapeutic performance.

**Fig. 7:**
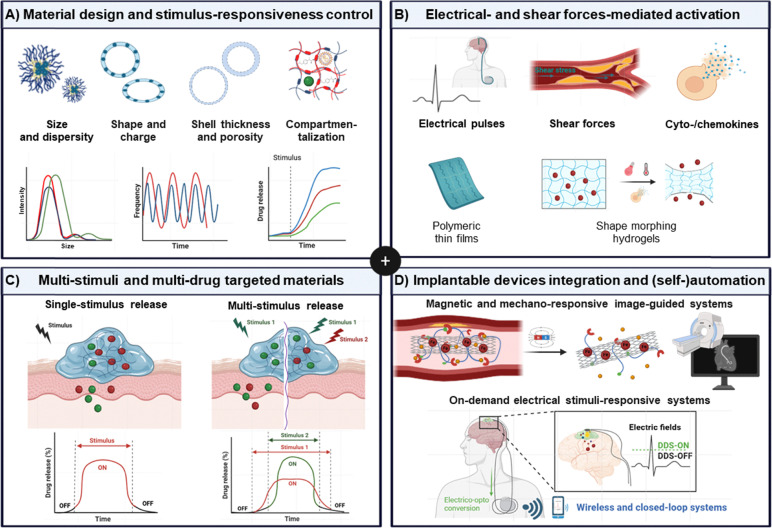
Design and engineering opportunities for stimuli-responsive therapeutic materials. Several engineering aspects can open new avenues for stimuli-responsive therapeutics, with their design and development driven by clear medical, disease, and patient needs. (A) Optimizing stimulus-responsiveness control in biologically relevant settings through precise material design, engineering, and tuning of physicochemical properties. (B) Exploring alternative physiological stimuli, including electrical pulses, shear forces, and cytokine/chemokine cues, enabling new therapeutic opportunities for neurological or cardiovascular diseases. (C) Developing multi-stimuli and multi-drug-containing systems, where single or combined triggers (*e.g.*, electrical, magnetic, pH) enable on–off drug release and therapy control. (D) Integration of stimuli-responsive therapeutics into implantable devices, incorporating magnetic, mechanical, and electrical responsiveness to support image-guided, on-demand, and closed-loop drug delivery.

From the materials research perspective, emerging concepts and future directions are focused on (a) optimizing material designs and structural engineering, improving stimulus-responsiveness control in biological settings; (b) exploring materials and technologies responsive to underexplored stimuli such as electrical pulses or shear forces, opening new opportunities to treat indications such as stroke or epilepsy; (c) triggering therapeutic activation and (multi)drug release *via* combined stimuli, including bidirectional on–off responsive structures; (d) evaluating their integration into implantable devices and vascular grafts for on-demand treatment control in regenerative medicine applications and incorporation into robotic systems to develop remote and self-activatable therapeutics (Fig. 7).

### 5.1. Material design and stimulus-responsiveness control

Promoting quality-by-design approaches of (novel) materials and improved control over the constituting building blocks and their properties (including underexplored engineering aspects such as microbubble shell thickness and porosity, or hydrogel compartmentalization)^53^ will open new avenues to more accurately construct nano- and microscale materials with enhanced control over specific stimuli (*e.g.*, narrow ultrasound frequencies). Advances in electroactive polymers and semiconductive membranes are already promoting better spatiotemporal control over stimuli activation due to the conversion of the electrical stimuli directly into mechanical motion, enabling fast, reversible, and spatially precise actuation.^264,265^ Shape memory hydrogels also offer enhanced responsiveness control due to their ability to undergo reversible, stimulus-specific deformation and then recover their original shape in response to environmental triggers.^266^

### 5.2. Electrical- and shear forces-mediated activation

While traditionally underexplored, electrical stimuli- and shear forces-responsive systems are gaining traction,^267^ particularly for neuromodulation (*e.g.*, epilepsy)^268^ or cardiovascular conditions (*e.g.*, clot-targeted therapies).^269^ Magneto-/electro-responsive polymers^270^ and mechano-sensitive hydrogels^271^ offer novel opportunities for precise, non-invasive control of therapeutic release due to shape-morphing capabilities under remote activation.

### 5.3. Multi-stimuli and multi-drug targeted materials

Drug delivery systems can be engineered to be triggered by different, combined stimuli (*e.g.*, pH and redox,^272^ photo and magnetic,^273^ or pH and temperature^216^) to improve drug targeting, and treatment specificity and control. Multi-drug nanomaterials can potentially achieve combined therapeutic effects and overcome drug resistance.^274^ Besides triggering co-delivery of synergistic drug combinations,^275^ responsiveness to multiple stimuli enables reversible on–off behavior governed by logic-gate-like mechanisms, where drug release occurs only when specific combinations of triggers are simultaneously present.^276^

### 5.4. Implantable devices integration and (self-)automation

The integration of stimuli-responsive materials into implantable systems allows for localized, sustained, and on-demand therapeutic delivery directly at the disease site, improving both efficacy and patient compliance. Implantable devices incorporating pH-, redox-, or enzyme-responsive hydrogels have been developed for post-surgical cancer therapy, offering controlled drug release in response to the tumor microenvironment's biochemical cues.^277^ Active implantable drug delivery systems, including soft robotics, represent a new generation of smart, stimuli-responsive implants capable of remotely controlled and autonomous therapeutic release, integrating powered actuators, control logic, and communication interfaces.^278,279^ For instance, soft implantable devices have recently been designed for seizure treatment, continuously monitoring electroencephalogram signals and using wireless power transmission to trigger drug release during emergencies.^280^ Photodynamic therapy can also benefit from implantable devices that use real-time monitoring and wireless power to intelligently modulate treatment based on optical or thermal feedback.^248,281,282^ Additionally, such systems can be combined with imaging platforms (*e.g.*, SPION),^283^ enabling real-time visualization of the implant or payload and its therapeutic activity *via* MRI, PET, or fluorescence imaging to guide and personalize treatments.

Progress across these areas will continue to expand the clinical impact of stimuli-responsive therapeutics and enhance their relevance in the treatment of other indications like neurological and cardiovascular diseases, wound healing, and regenerative medicine.

While hurdles remain, the field is entering a critical phase where carefully elaborated material design needs to be ensured, primarily motivated by a disease- and medical need-driven mindset, and supported by robust translational frameworks, to increase the translational success rate of stimuli-responsive (nano- and macroscale) therapeutics and broaden their clinical impact.

## Conflicts of interest

The authors declare that they have no known competing financial interests or personal relationships that could have appeared to influence the work reported in this paper.

## Supplementary Material

CS-055-D6CS00165C-s001

## Data Availability

The data supporting this article have been included as part of the supplementary information (SI). Keyword search scheme is presented in SI. See DOI: https://doi.org/10.1039/d6cs00165c.

## References

[cit1] Baskaran R., Lee J., Yang S.-G. (2018). Biomater. Res..

[cit2] Da Silva J., Bienassis C., Schmitt P., Berjaud C., Guedj M., Paris S. (2024). J. Exp. Clin. Cancer Res..

[cit3] Gerken L. R. H., Gerdes M. E., Pruschy M., Herrmann I. K. (2023). Mater. Horizons.

[cit4] Correia J. H., Rodrigues J. A., Pimenta S., Dong T., Yang Z. (2021). Pharmaceutics.

[cit5] Zhao X., Liu J., Fan J., Chao H., Peng X. (2021). Chem. Soc. Rev..

[cit6] Zhang Z., Du Y., Shi X., Wang K., Qu Q., Liang Q., Ma X., He K., Chi C., Tang J., Liu B., Ji J., Wang J., Dong J., Hu Z., Tian J. (2024). Nat. Rev. Clin. Oncol..

[cit7] Ashrafizadeh M., Delfi M., Zarrabi A., Bigham A., Sharifi E., Rabiee N., Paiva-Santos A. C., Kumar A. P., Tan S. C., Hushmandi K., Ren J., Zare E. N., Makvandi P. (2022). J. Controlled Release.

[cit8] Yu Y., Cheng Y., Tong J., Zhang L., Wei Y., Tian M. (2021). J. Mater. Chem. B.

[cit9] Molinaro M., Skrodzki D., Pan D. (2024). Wiley Interdiscip. Rev.:Nanomed. Nanobiotechnol..

[cit10] Rezaei B., Yari P., Sanders S. M., Wang H., Chugh V. K., Liang S., Mostufa S., Xu K., Wang J., Gómez-Pastora J., Wu K. (2024). Small.

[cit11] Włodarczyk A., Gorgoń S., Radoń A., Bajdak-Rusinek K. (2022). Nanomaterials.

[cit12] Shakeri-Zadeh A., Bulte J. W. M. (2025). Nat. Rev. Bioeng..

[cit13] Zhu K., Wang J., Wang Z., Chen Q., Song J., Chen X. (2025). Angew. Chem., Int. Ed..

[cit14] Li D., Yang Y., Li D., Pan J., Chu C., Liu G. (2021). Small.

[cit15] Ding H., Tan P., Fu S., Tian X., Zhang H., Ma X., Gu Z., Luo K. (2022). J. Controlled Release.

[cit16] Fu Z., Li S., Han S., Shi C., Zhang Y. (2022). Signal Transduction Targeted Ther..

[cit17] Meng X., Shen Y., Zhao H., Lu X., Wang Z., Zhao Y. (2024). J. Nanobiotechnol..

[cit18] Wei Y., Lv J., Zhu S., Wang S., Su J., Xu C. (2024). Drug Discovery Today.

[cit19] Dumontet C., Reichert J. M., Senter P. D., Lambert J. M., Beck A. (2023). Nat. Rev. Drug Discovery.

[cit20] Bhatia S. N., Chen X., Dobrovolskaia M. A., Lammers T. (2022). Nat. Rev. Cancer.

[cit21] Mura S., Nicolas J., Couvreur P. (2013). Nat. Mater..

[cit22] Lammers T. (2024). Adv. Mater..

[cit23] Grzybowski A., Pietrzak K. (2012). Clin. Dermatol..

[cit24] Szeimies R.-M., Dirschka T., Fargnoli M. C., Gilaberte Y., Hædersdal M., Chavda R., Calzavara-Pinton P. (2023). Dermatol. Ther..

[cit25] Fabre M., Mateo L., Lamaa D., Baillif S., Pagès G., Demange L., Ronco C., Benhida R. (2022). Molecules.

[cit26] Photodynamic Therapy Market Size And Forecast, https://www.verifiedmarketresearch.com/product/photodynamic-therapy-market/, (accessed October 2025)

[cit27] Photodynamic Therapy Market Size and Forecast 2025 to 2034, https://www.precedenceresearch.com/photodynamic-therapy-market, (accessed October 2025)

[cit28] Zhang Y., Zhang S., Zhang Z., Ji L., Zhang J., Wang Q., Guo T., Ni S., Cai R., Mu X., Long W., Wang H. (2021). Front. Chem..

[cit29] Kobayashi H., Choyke P. L., Ogawa M. (2023). Curr. Opin. Chem. Biol..

[cit30] Kadria-Vili Y., Schwartz J. A., Polascik T. J., Goodrich G. P., Jorden D., Pinder D., Halas N. J., Rastinehad A. R. (2024). Nanomaterials.

[cit31] Chaudhry M., Lyon P., Coussios C., Carlisle R. (2022). Expert Opin. Drug Delivery.

[cit32] Lawrie A., Brisken A., Francis S., Cumberland D., Crossman D., Newman C. (2000). Gene Ther..

[cit33] Rodriguez B., Rivera D., Zhang J. Y., Brown C., Young T., Williams T., Huq S., Mattioli M., Bouras A., Hadjpanayis C. G. (2024). Pharmaceuticals.

[cit34] Schwake M., Müther M., Bruns A.-K., Zinnhardt B., Warneke N., Holling M., Schipmann S., Brokinkel B., Wölfer J., Stummer W., Grauer O. (2022). Cancers.

[cit35] Soetaert F., Korangath P., Serantes D., Fiering S., Ivkov R. (2020). Adv. Drug Delivery Rev..

[cit36] Caculitan N. G., dela J., Chuh C., Ma Y., Zhang D., Kozak K. R., Liu Y., Pillow T. H., Sadowsky J., Cheung T. K., Phung Q., Haley B., Lee B. C., Akita R. W., Sliwkowski M. X., Polson A. G. (2017). Cancer Res..

[cit37] Antibody Drug Conjugates Market Size, Share and Trends 2026 to 2035, https://www.precedenceresearch.com/antibody–drug-conjugates-market, (accessed October 2025)

[cit38] Yang J., Kopeček J. (2017). Curr. Opin. Colloid Interface Sci..

[cit39] Zheng J., Song X., Yang Z., Yin C., Luo W., Yin C., Ni Y., Wang Y., Zhang Y. (2022). J. Controlled Release.

[cit40] Peña Q., Wang A., Zaremba O., Shi Y., Scheeren H. W., Metselaar J. M., Kiessling F., Pallares R. M., Wuttke S., Lammers T. (2022). Chem. Soc. Rev..

[cit41] Su Z., Xiao D., Xie F., Liu L., Wang Y., Fan S., Zhou X., Li S. (2021). Acta Pharm. Sin. B.

[cit42] Song K., Jiang C., Huang S., Li X. (2025). Mater. Chem. Front..

[cit43] Zong Y., Lin Y., Wei T., Cheng Q. (2023). Adv. Mater..

[cit44] Maboudi A. H., Lotfipour M. H., Rasouli M., Azhdari M. H., MacLoughlin R., Bekeschus S., Doroudian M. (2024). Nanotechnol. Rev..

[cit45] Cabral H., Miyata K., Osada K., Kataoka K. (2018). Chem. Rev..

[cit46] Javia A., Vanza J., Bardoliwala D., Ghosh S., Misra L. A., Patel M., Thakkar H. (2022). Int. J. Pharm..

[cit47] Barmin R. A., Moosavifar M., Dasgupta A., Herrmann A., Kiessling F., Pallares R. M., Lammers T. (2023). Chem. Sci..

[cit48] Tian B., Hua S., Liu J. (2023). Carbohydr. Polym..

[cit49] Petrovici A. R., Pinteala M., Simionescu N. (2023). Molecules.

[cit50] Hosseini S. M., Mohammadnejad J., Najafi-Taher R., Zadeh Z. B., Tanhaei M., Ramakrishna S. (2023). ACS Appl. Bio Mater..

[cit51] Koohi Moftakhari Esfahani M., Alavi S. E., Cabot P. J., Islam N., Izake E. L. (2022). Pharmaceutics.

[cit52] Devi S., Kumar M., Tiwari A., Tiwari V., Kaushik D., Verma R., Bhatt S., Sahoo B. M., Bhattacharya T., Alshehri S., Ghoneim M. M., Babalghith A. O., Batiha G. E.-S. (2022). Front. Mater..

[cit53] Moosavifar M., Barmin R. A., Rama E., Rix A., Gumerov R. A., Lisson T., Bastard C., Rütten S., Avraham-Radermacher N., Koehler J., Pohl M., Kulkarni V., Baier J., Koletnik S., Zhang R., Dasgupta A., Motta A., Weiler M., Potemkin I. I., Schmitz G., Kiessling F., Lammers T., Pallares R. M. (2024). Adv. Sci..

[cit54] Dasgupta A., Sun T., Palomba R., Rama E., Zhang Y., Power C., Moeckel D., Liu M., Sarode A., Weiler M., Motta A., Porte C., Magnuska Z., Said Elshafei A., Barmin R., Graham A., McClelland A., Rommel D., Stickeler E., Kiessling F., Pallares R. M., De Laporte L., Decuzzi P., McDannold N., Mitragotri S., Lammers T. (2023). Proc. Natl. Acad. Sci. U. S. A..

[cit55] Hsu J. C., Tang Z., Eremina O. E., Sofias A. M., Lammers T., Lovell J. F., Zavaleta C., Cai W., Cormode D. P. (2023). Nat. Rev. Methods Primers.

[cit56] Zhang R., Kiessling F., Lammers T., Pallares R. M. (2023). Drug Delivery Transl. Res..

[cit57] Yu S., Xia G., Yang N., Yuan L., Li J., Wang Q., Li D., Ding L., Fan Z., Li J. (2024). Int. J. Mol. Sci..

[cit58] Dadfar S. M., Roemhild K., Drude N. I., von Stillfried S., Knüchel R., Kiessling F., Lammers T. (2019). Adv. Drug Delivery Rev..

[cit59] Lux F., Tran V. L., Thomas E., Dufort S., Rossetti F., Martini M., Truillet C., Doussineau T., Bort G., Denat F., Boschetti F., Angelovski G., Detappe A., Crémillieux Y., Mignet N., Doan B.-T., Larrat B., Meriaux S., Barbier E., Roux S., Fries P., Müller A., Abadjian M.-C., Anderson C., Canet-Soulas E., Bouziotis P., Barberi-Heyob M., Frochot C., Verry C., Balosso J., Evans M., Sidi-Boumedine J., Janier M., Butterworth K., McMahon S., Prise K., Aloy M.-T., Ardail D., Rodriguez-Lafrasse C., Porcel E., Lacombe S., Berbeco R., Allouch A., Perfettini J.-L., Chargari C., Deutsch E., Le Duc G., Tillement O. (2018). Br. J. Radiol..

[cit60] Barth R. F., Mi P., Yang W. (2018). Cancer Commun..

[cit61] Xue L., Thatte A. S., Mai D., Haley R. M., Gong N., Han X., Wang K., Sheppard N. C., June C. H., Mitchell M. J. (2024). Nat. Rev. Mater..

[cit62] Gong L., Zhang Y., Liu C., Zhang M., Han S. (2021). Int. J. Nanomed..

[cit63] Sharma K. K. K., Swarts S. G., Bernhard W. A. (2011). J. Phys. Chem. B.

[cit64] Zhang Y.-F., Lu M. (2024). Front. Bioeng. Biotechnol..

[cit65] Anand S., Chan T. A., Hasan T., Maytin E. V. (2021). Pharmaceuticals.

[cit66] Parshad B., Arora S., Singh B., Pan Y., Tang J., Hu Z., Patra H. K. (2025). Commun. Chem..

[cit67] Zahnreich S., Bhatti A., Ahmad B., Drabke S., Kaufmann J., Schmidberger H. (2025). Cells.

[cit68] Dou Y., Hynynen K., Allen C. (2017). J. Controlled Release.

[cit69] Celsion Corporation Receives Recommendation from Independent Data Monitoring Committee to Consider Stopping the Phase III OPTIMA Study, https://investor.celsion.com/news-releases/news-release-details/celsion-corporation-receives-recommendation-independent-data, (accessed December 2025)

[cit70] Fralish Z., Chen A., Khan S., Zhou P., Reker D. (2024). Nat. Rev. Drug Discovery.

[cit71] Rautio J., Meanwell N. A., Di L., Hageman M. J. (2018). Nat. Rev. Drug Discovery.

[cit72] Kobayashi H., Furusawa A., Rosenberg A., Choyke P. L. (2021). Int. Immunol..

[cit73] Kato T., Okada R., Furusawa A., Inagaki F., Wakiyama H., Furumoto H., Okuyama S., Fukushima H., Choyke P. L., Kobayashi H. (2021). Mol. Cancer Ther..

[cit74] Kobzev D., Semenova O., Tatarets A., Bazylevich A., Gellerman G., Patsenker L. (2023). Dyes Pigm..

[cit75] Worley B., Harikumar V., Reynolds K., Dirr M. A., Christensen R. E., Anvery N., Yi M. D., Poon E., Alam M. (2023). Arch. Dermatol. Res..

[cit76] Karges J. (2022). Angew. Chem., Int. Ed..

[cit77] Semenova O., Kobzev D., Hovor I., Atrash M., Nakonechny F., Kulyk O., Bazylevich A., Gellerman G., Patsenker L. (2023). Pharmaceutics.

[cit78] Hu X., Fang Z., Zhu C., Yang Y., Yang Z., Huang W. (2024). Adv. Funct. Mater..

[cit79] Chowdhury P., Chan Y.-H. (2022). Mol. Syst. Des. Eng..

[cit80] Zhang Y., Li S., Fang X., Miao B., Wang Y., Liu J., Nie G., Zhang B. (2022). Nanophotonics.

[cit81] Yukawa H., Sato K., Baba Y. (2023). Adv. Drug Delivery Rev..

[cit82] Pallares R. M., Kiessling F., Lammers T. (2024). Nanomedicine.

[cit83] Yang Y., Jiang S., Stanciu S. G., Peng H., Wu A., Yang F. (2024). Mater. Horizons.

[cit84] Lopez-Beltran A., Cookson M. S., Guercio B. J., Cheng L. (2024). BMJ.

[cit85] Mitragotri S. (2005). Nat. Rev. Drug Discovery.

[cit86] Meng Y., Hynynen K., Lipsman N. (2021). Nat. Rev. Neurol..

[cit87] Stride E., Coussios C. (2019). Nat. Rev. Phys..

[cit88] Stride E., Segers T., Lajoinie G., Cherkaoui S., Bettinger T., Versluis M., Borden M. (2020). Ultrasound Med. Biol..

[cit89] Lentacker I., De Cock I., Deckers R., De Smedt S. C., Moonen C. T. W. (2014). Adv. Drug Delivery Rev..

[cit90] Cattaneo M., Guerriero G., Shakya G., Krattiger L. A., Paganella L. G., Narciso M. L., Supponen O. (2025). Nat. Phys..

[cit91] Carpentier A., Canney M., Vignot A., Reina V., Beccaria K., Horodyckid C., Karachi C., Leclercq D., Lafon C., Chapelon J.-Y., Capelle L., Cornu P., Sanson M., Hoang-Xuan K., Delattre J.-Y., Idbaih A. (2016). Sci. Transl. Med..

[cit92] Ahluwalia M., McDermott M., Burns T., de Groot J., Mogilner A., Achrol A., Shah B., Bettegowda C., Ozair A., Khosla A., Schwartz T., Sahgal A., Mishra M., Everson R., Weinberg J., Amankulor N., Sporrer J., Cifarelli C., Rezai A., Lipsman N., Woodworth G. F. (2023). Neuro-Oncol..

[cit93] Dimcevski G., Kotopoulis S., Bjånes T., Hoem D., Schjøtt J., Gjertsen B. T., Biermann M., Molven A., Sorbye H., McCormack E., Postema M., Gilja O. H. (2016). J. Controlled Release.

[cit94] Lipsman N., Meng Y., Bethune A. J., Huang Y., Lam B., Masellis M., Herrmann N., Heyn C., Aubert I., Boutet A., Smith G. S., Hynynen K., Black S. E. (2018). Nat. Commun..

[cit95] Kiessling F., Fokong S., Koczera P., Lederle W., Lammers T. (2012). J. Nucl. Med..

[cit96] Fokong S., Theek B., Wu Z., Koczera P., Appold L., Jorge S., Resch-Genger U., van Zandvoort M., Storm G., Kiessling F., Lammers T. (2012). J. Controlled Release.

[cit97] Koczera P., Appold L., Shi Y., Liu M., Dasgupta A., Pathak V., Ojha T., Fokong S., Wu Z., van Zandvoort M., Iranzo O., Kuehne A. J. C., Pich A., Kiessling F., Lammers T. (2017). J. Controlled Release.

[cit98] Nittayacharn P., Abenojar E., Cooley M. B., Berg F. M., Counil C., Sojahrood A. J., Khan M. S., Yang C., Berndl E., Golczak M., Kolios M. C., Exner A. A. (2024). J. Controlled Release.

[cit99] Cheng Y., Cheng H., Jiang C., Qiu X., Wang K., Huan W., Yuan A., Wu J., Hu Y. (2015). Nat. Commun..

[cit100] Huang S. L. (2010). Methods Mol. Biol..

[cit101] Purohit M. P., Yu B. J., Roy K. S., Xiang Y., Ewbank S. N., Azadian M. M., Hart A. R., Muwanga G. P. B., Martinez P. J., Wang J. B., Taoube A. K., Markarian E., Macedo N., Kwan A. K., Lopez D. G., Airan R. D. (2025). Nat. Nanotechnol..

[cit102] Kobzev D., Semenova O., Aviel-Ronen S., Kulyk O., Carmieli R., Mirzabekov T., Gellerman G., Patsenker L. (2024). Int. J. Mol. Sci..

[cit103] Rosenthal I., Sostaric J. Z., Riesz P. (2004). Ultrason. Sonochem..

[cit104] McMahon S. J., Hyland W. B., Muir M. F., Coulter J. A., Jain S., Butterworth K. T., Schettino G., Dickson G. R., Hounsell A. R., O’Sullivan J. M., Prise K. M., Hirst D. G., Currell F. J. (2011). Radiother. Oncol..

[cit105] Schuemann J., Berbeco R., Chithrani D. B., Cho S. H., Kumar R., McMahon S. J., Sridhar S., Krishnan S. (2016). Int. J. Radiat. Oncol., Biol., Phys..

[cit106] Taheri A., Khandaker M. U., Moradi F., Bradley D. A. (2023). Radiat. Phys. Chem..

[cit107] Jackson N., Cecchi D., Beckham W., Chithrani D. B. (2024). Molecules.

[cit108] Qi P., Chen Q., Tu D., Yao S., Zhang Y., Wang J., Xie C., Pan C., Peng H. (2020). Biomater. Sci..

[cit109] Li L., Wang M., Zhao Q., Bai P., Hao H., Zhang Z., Liu T., Yang Y., Pu K., Zhang R. (2025). Angew. Chem., Int. Ed..

[cit110] Performance and Safety of thermotherapy with nanoparticles (NanoTherm® Therapy System) as an adjuvant therapy to the Standard of Care treatment of patients with recurrent glioblastoma, https://drks.de/search/en/trial/DRKS00023339/details, (accessed December 2025)

[cit111] Rezaee M., Alizadeh E., Cloutier P., Hunting D. J., Sanche L. (2014). ChemMedChem.

[cit112] Teixeira P. C. N. , Cytologic and radiosensibilizer action of the methylene blue, Biophysics Institute Carlos Chagas Filho, Universidade Federal, Rio de Janeiro (Brazil), 1989

[cit113] Yu X., Liu B., Zhang N., Wang Q., Cheng G. (2021). Front. Cell Dev. Biol..

[cit114] You Y., Wen R., Pathak R., Li A., Li W., St Clair D., Hauer-Jensen M., Zhou D., Liang Y. (2014). Cell Death Dis..

[cit115] Rachamala H. K., Madamsetty V. S., Angom R. S., Nakka N. M., Dutta S. K., Wang E., Mukhopadhyay D., Pal K. (2024). J. Exp. Clin. Cancer Res..

[cit116] Leo S., Gutierrez N. M. C., Bulin A.-L., Coll J. L., Sancey L., Habermeyer B., Broekgaarden M. (2025). Eur. J. Med. Chem..

[cit117] Adams S. R., Yang H. C., Savariar E. N., Aguilera J., Crisp J. L., Jones K. A., Whitney M. A., Lippman S. M., Cohen E. E. W., Tsien R. Y., Advani S. J. (2016). Nat. Commun..

[cit118] Ni K., Lan G., Chan C., Quigley B., Lu K., Aung T., Guo N., La Riviere P., Weichselbaum R. R., Lin W. (2018). Nat. Commun..

[cit119] Chen Z., Han F., Du Y., Shi H., Zhou W. (2023). Signal Transduction Targeted Ther..

[cit120] Li X., Wang H., Li Z., Tao F., Wu J., Guan W., Liu S. (2023). Front. Oncol..

[cit121] Li Q., Kartikowati C. W., Horie S., Ogi T., Iwaki T., Okuyama K. (2017). Sci. Rep..

[cit122] Li Y., Zhang R., Barmin R., Rama E., Schoenen M., Schrank F., Schulz V., Slabu I., Kiessling F., Lammers T., Pallares R. M. (2024). Nanoscale Adv..

[cit123] Sahoo P., Choudhary P., Laha S. S., Dixit A., Mefford O. T. (2023). Chem. Commun..

[cit124] Maier A., Jia Q., Shukla K., Dugulan A. I., Hagedoorn P.-L., van Oossanen R., van Rhoon G., Denkova A. G., Djanashvili K. (2024). ACS Appl. Nano Mater..

[cit125] Singh P., Duraisamy K., Raitmayr C., Sharma K. S., Korzun T., Singh K., Moses A. S., Yamada K., Grigoriev V., Demessie A. A., Park Y., Goo Y. T., Mamnoon B., Souza A. P. M., Michimoto K., Farsad K., Jaiswal A., Taratula O. R., Taratula O. (2025). Adv. Funct. Mater..

[cit126] Araújo E. V., Carneiro S. V., Neto D. M. A., Freire T. M., Costa V. M., Freire R. M., Fechine L. M. U. D., Clemente C. S., Denardin J. C., dos Santos J. C. S., Santos-Oliveira R., Rocha J. S., Fechine P. B. A. (2024). Adv. Colloid Interface Sci..

[cit127] Patri S., Thanh N. T. K., Kamaly N. (2024). Nanoscale.

[cit128] Talelli M., Rijcken C. J. F., Lammers T., Seevinck P. R., Storm G., van Nostrum C. F., Hennink W. E. (2009). Langmuir.

[cit129] Vlasova K. Y., Piroyan A., Le-Deygen I. M., Vishwasrao H. M., Ramsey J. D., Klyachko N. L., Golovin Y. I., Rudakovskaya P. G., Kireev I. I., Kabanov A. V., Sokolsky-Papkov M. (2019). J. Colloid Interface Sci..

[cit130] Rose J. C., Cámara-Torres M., Rahimi K., Köhler J., Möller M., De Laporte L. (2017). Nano Lett..

[cit131] Qian B., Shen A., Huang S., Shi H., Long Q., Zhong Y., Qi Z., He X., Zhang Y., Hai W., Wang X., Cui Y., Chen Z., Xuan H., Zhao Q., You Z., Ye X. (2023). Adv. Sci..

[cit132] Karimi M., Sahandi Zangabad P., Ghasemi A., Amiri M., Bahrami M., Malekzad H., Ghahramanzadeh Asl H., Mahdieh Z., Bozorgomid M., Ghasemi A., Rahmani Taji Boyuk M. R., Hamblin M. R. (2016). ACS Appl. Mater. Interfaces.

[cit133] Khan B., Arbab A., Khan S., Fatima H., Bibi I., Chowdhry N. P., Ansari A. Q., Ursani A. A., Kumar S., Hussain J., Abdullah S. (2023). MedComm: Biomater. Appl..

[cit134] Abuwatfa W. H., Awad N. S., Pitt W. G., Husseini G. A. (2022). Polymers.

[cit135] Shriky B., Kelly A., Isreb M., Babenko M., Mahmoudi N., Rogers S., Shebanova O., Snow T., Gough T. (2020). J. Colloid Interface Sci..

[cit136] Giugliano F., Corti C., Tarantino P., Michelini F., Curigliano G. (2022). Curr. Oncol. Rep..

[cit137] Kobzev D., Prasad C., Walunj D., Gotman H., Semenova O., Bazylevich A., Patsenker L., Gellerman G. (2023). Eur. J. Med. Chem..

[cit138] Zhou D., Zhai X., Zhang L., Xie Z., Wang Y., Zhen Y., Gao R., Miao Q. (2024). npj Precis. Oncol..

[cit139] Tsuchikama K., Anami Y., Ha S. Y. Y., Yamazaki C. M. (2024). Nat. Rev. Clin. Oncol..

[cit140] Mullard A. (2025). Nat. Rev. Drug Discovery.

[cit141] Ma W., Wang X., Zhang D., Mu X. (2024). Int. J. Nanomed..

[cit142] Sonju J. J., Dahal A., Singh S. S., Gu X., Johnson W. D., Muthumula C. M. R., Meyer S. A., Jois S. D. (2022). Int. J. Pharm..

[cit143] Suzuki Y., Ishihara H. (2021). Drug Metab. Pharmacokinet..

[cit144] Klipp A., Burger M., Leroux J.-C. (2023). Adv. Drug Delivery Rev..

[cit145] Aryal S., Hu C.-M. J., Zhang L. (2010). ACS Nano.

[cit146] Chida T., Miura Y., Cabral H., Nomoto T., Kataoka K., Nishiyama N. (2018). J. Controlled Release.

[cit147] Yi X.-Q., Zhang Q., Zhao D., Xu J.-Q., Zhong Z.-L., Zhuo R.-X., Li F. (2016). Polym. Chem..

[cit148] Egorova V. S., Kolesova E. P., Lopus M., Yan N., Parodi A., Zamyatnin A. A. (2023). Pharmaceutics.

[cit149] Son J., Parveen S., MacPherson D., Marciano Y., Huang R. H., Ulijn R. V. (2023). Biomater. Sci..

[cit150] Parshad B., Baker A. G., Ahmed I., Estepa-Fernández A., Muñoz-Espín D., Fruk L. (2025). Small.

[cit151] Pallares R. M., Barmin R. A., Wang A., Kiessling F., Lammers T. (2025). J. Controlled Release.

[cit152] Manenti G., Perretta T., Nezzo M., Fraioli F. R., Carreri B., Gigliotti P. E., Micillo A., Malizia A., Di Giovanni D., Ryan C. P., Garaci F. G. (2024). Cancers.

[cit153] Jung H. S., Kim H. J. (2022). World J. Surg. Oncol..

[cit154] Li Y., Li Y., Song Y., Liu S. (2024). Oncol. Rep..

[cit155] Gibault F., Bailly F., Corvaisier M., Coevoet M., Huet G., Melnyk P., Cotelle P. (2017). ChemMedChem.

[cit156] Alnaieb Z., Osman E., Medani S. (2025). Urol. Ann..

[cit157] Dias M., Zhang R., Lammers T., Pallares R. M. (2025). Drug Delivery Transl. Res..

[cit158] Cognetti D. M., Johnson J. M., Curry J. M., Kochuparambil S. T., McDonald D., Mott F., Fidler M. J., Stenson K., Vasan N. R., Razaq M. A., Campana J., Ha P., Mann G., Ishida K., Garcia-Guzman M., Biel M., Gillenwater A. M. (2021). Head Neck.

[cit159] Gomes-da-Silva L. C., Kepp O., Kroemer G. (2020). Oncoimmunology.

[cit160] Miyazaki N. L., Furusawa A., Choyke P. L., Kobayashi H. (2023). Cancers.

[cit161] Guo Y., Shi M., Yang A., Feng J., Zhu X., Choi Y., Hu G., Pan J., Hu C., Luo R., Zhang Y., Zhou L., Cheng Y., Lüpfert C., Cai J., Shi Y. (2015). Head Neck.

[cit162] Theralase(R) Provides Update on Phase II Bladder Cancer Clinical Study, https://www.biospace.com/theralase-r-provides-update-on-phase-ii-bladder-cancer-clinical-study, (accessed November 2025)

[cit163] Balar A. V., Kamat A. M., Kulkarni G. S., Uchio E. M., Boormans J. L., Roumiguié M., Krieger L. E. M., Singer E. A., Bajorin D. F., Grivas P., Seo H. K., Nishiyama H., Konety B. R., Li H., Nam K., Kapadia E., Frenkl T., de Wit R. (2021). Lancet Oncol..

[cit164] Ruvidar Demonstrates Potential for Durable Responses in Non–Muscle Invasive Bladder Cancer, https://www.onclive.com/view/ruvidar-demonstrates-potential-for-durable-responses-in-non-muscle-invasive-bladder-cancer, (accessed November 2025)

[cit165] Lin A. Y., Yang E., Rink J. S., Xu D., Miller S., Gordon L. I. (2023). Blood.

[cit166] Paefgen V., Doleschel D., Kiessling F. (2015). Front. Pharmacol..

[cit167] Guo Y., Lee H., Kim C., Park C., Yamamichi A., Chuntova P., Gallus M., Bernabeu M. O., Okada H., Jo H., Arvanitis C. (2024). Nat. Commun..

[cit168] Borden M. A., Kruse D. E., Caskey C. F., Zhao S., Dayton P. A., Ferrara K. W. (2005). IEEE Trans. Ultrason. Ferroelectr. Freq. Control.

[cit169] Rajora M. A., Dhaliwal A., Zheng M., Choi V., Overchuk M., Lou J. W. H., Pellow C., Goertz D., Chen J., Zheng G. (2024). Adv. Sci..

[cit170] Shin J., Kong C., Cho J. S., Lee J., Koh C. S., Yoon M.-S., Na Y. C., Chang W. S., Chang J. W. (2018). Neurosurg. Focus.

[cit171] Dasgupta A., Liu M., Ojha T., Storm G., Kiessling F., Lammers T. (2016). Drug Discovery Today Technol..

[cit172] Wu C.-C., Szalontay L., Pouliopoulos A. N., Bae S., Berg X., Wei H.-J., Webster Carrion A., Kokossis D., Sethi C., Fino J., Shatravka H., Lipina J., Ji R., Liu K., Yousefian O., Gallitto M., Yoh N., Englander Z., McQuillan N., Tazhibi M., De Los Santos G., Canoll P., Jin Z., Garvin J., Gartrell R. D., Pavisic J., Maddocks A., Lignelli A., Feldstein N., Konofagou E. E., Zacharoulis S. (2025). Sci. Transl. Med..

[cit173] Moore-Palhares D., Dasgupta A., Saifuddin M., Anzola Pena M. L., Prasla S., Ho L., Lu L., Kung J., McNabb E., Sannachi L., Vesprini D., Chen H., Karam I., Soliman H., Szumacher E., Chow E., Gandhi S., Trudeau M., Curpen B., Stanisz G. J., Kolios M., Czarnota G. J. (2024). PLoS Med..

[cit174] Moore-Palhares D., Saifuddin M., Dasgupta A., Anzola Pena M. L., Prasla S., Ho L., Lu L., Kung J., Karam I., Poon I., Bayley A., McNabb E., Stanisz G., Kolios M., Czarnota G. J. (2024). Radiother. Oncol..

[cit175] Stummer W., Novotny A., Stepp H., Goetz C., Bise K., Reulen H. J. (2000). J. Neurosurg..

[cit176] Hadjipanayis C. G., Stummer W. (2019). J. Neurooncol..

[cit177] Sonabend A. M., Gould A., Amidei C., Ward R., Schmidt K. A., Zhang D. Y., Gomez C., Bebawy J. F., Liu B. P., Bouchoux G., Desseaux C., Helenowski I. B., Lukas R. V., Dixit K., Kumthekar P., Arrieta V. A., Lesniak M. S., Carpentier A., Zhang H., Muzzio M., Canney M., Stupp R. (2023). Lancet Oncol..

[cit178] Carpentier A., Stupp R., Sonabend A. M., Dufour H., Chinot O., Mathon B., Ducray F., Guyotat J., Baize N., Menei P., de Groot J., Weinberg J. S., Liu B. P., Guemas E., Desseaux C., Schmitt C., Bouchoux G., Canney M., Idbaih A. (2024). Nat. Commun..

[cit179] Haram M., Hansen R., Myhre O. F., Solberg S., Amini N., Angelsen B. A., de C., Davies L., Hofsli E. (2025). WFUMB Ultrasound Open.

[cit180] Han F., Wang Y., Dong X., Lin Q., Wang Y., Gao W., Yun M., Li Y., Gao S., Huang H., Li N., Luo T., Luo X., Qiu M., Zhang D., Yan K., Li A., Liu Z. (2023). Eur. Radiol..

[cit181] Rix A., Piepenbrock M., Flege B., von Stillfried S., Koczera P., Opacic T., Simons N., Boor P., Thoröe-Boveleth S., Deckers R., May J.-N., Lammers T., Schmitz G., Stickeler E., Kiessling F. (2021). Theranostics.

[cit182] Sun X., Guo S., Yao J., Wang H., Peng C., Li B., Wang Y., Jiang Y., Wang T., Yang Y., Cheng J., Wang W., Cao Z., Zhao X., Li X., Sun J., Yang J., Tian F., Chen X., Li Q., Gao W., Shen J., Zhou Q., Wang P., Li Z., Tian Z., Zhang Z., Cao W., Li M., Tian Y. (2019). Cardiovasc. Res..

[cit183] Jiang Y., Fan J., Li Y., Wu G., Wang Y., Yang J., Wang M., Cao Z., Li Q., Wang H., Zhang Z., Wang Y., Li B., Sun F., Zhang H., Zhang Z., Li K., Tian Y. (2021). Int. J. Cardiol..

[cit184] De Felice F., Belgioia L., Alterio D., Bonomo P., Maddalo M., Paiar F., Denaro N., Corvò R., Merlotti A., Bossi P., Pappagallo G. L., D'Angelillo R. M., Magrini S. M., Arcangeli S. (2021). Crit. Rev. Oncol. Hematol..

[cit185] Łazar-Poniatowska M., Bandura A., Dziadziuszko R., Jassem J. (2021). Transl. Lung Cancer Res..

[cit186] Liu Y., Bao Y., Yang X., Sun S., Yuan M., Ma Z., Zhang W., Zhai Y., Wang Y., Men Y., Qin J., Xue L., Wang J., Hui Z. (2023). Front. Immunol..

[cit187] Liu S., Jiang T., Xiao L., Yang S., Liu Q., Gao Y., Chen G., Xiao W. (2021). Oncologist.

[cit188] Kurdi M., Alkhotani A., Alsinani T., Alkhayyat S., Katib Y., Jastaniah Z., Sabbagh A. J., Butt N. S., Toonsi F. A., Alharbi M., Baeesa S. (2025). Clin. Oncol..

[cit189] Pesce C., Rodella G., Fragassi A., Garofalo M., Salmaso S., Caliceti P., Gallez B., Malfanti A. (2025). Nanomedicine.

[cit190] Liu X., Zhang Y., Yang K.-Y., Zhang N., Jin F., Zou G.-R., Zhu X.-D., Xie F.-Y., Liang X.-Y., Li W.-F., He Z.-Y., Chen N.-Y., Hu W.-H., Wu H.-J., Shi M., Zhou G.-Q., Mao Y.-P., Guo R., Sun R., Huang J., Liang S.-Q., Wu W.-L., Su Z., Li L., Ai P., He Y.-X., Zang J., Chen L., Lin L., Huang S. H., Xu C., Lv J.-W., Li Y.-Q., Hong S.-B., Jie Y.-S., Li H., Huang S.-W., Liang Y.-L., Wang Y.-Q., Peng Y.-L., Zhu J.-H., Zang S.-B., Liu S.-R., Lin Q.-G., Li H.-J., Tian L., Liu L.-Z., Zhao H.-Y., Lin A.-H., Li J.-B., Liu N., Tang L.-L., Chen Y.-P., Sun Y., Ma J. (2024). Lancet.

[cit191] Machiels J.-P., Tao Y., Licitra L., Burtness B., Tahara M., Rischin D., Alves G., Lima I. P. F., Hughes B. G. M., Pointreau Y., Aksoy S., Laban S., Greil R., Burian M., Hetnał M., Delord J.-P., Mesía R., Taberna M., Waldron J. N., Simon C., Grégoire V., Harrington K. J., Swaby R. F., Zhang Y., Gumuscu B., Bidadi B., Siu L. L., Rischin D., Hughes B. G., Gao B., McGrath M., Greil R., Thurnher D., Fuereder T., Burian M., Rottey S., Machiels J.-P., Clement P. M., Henry S., Deheneffe S., Vasconcelos Alves G., Lima I. P. F., Mourão Dias J., De Marchi P. R. M., Mak M. P., Pereira de Santana Gomes A. J., Oliveira de Castro Junior D., Motta T. C., Agostinho Padoan M. L., Victorina A. P., de Azevedo S. J., Siu L. L., Brule S., Hilton J., Wang C. S., Bouganim N., Webster M., Walker J., Chua N., Zambrano A. R., Quiroga Echeverri A., Niño Gomez O. M., Ortiz C. A., Rojas L., Cardona Zorilla A., Urrego Meléndez O. M., Holečková P. B., Melichar B., Cvek J., Prausová J., Vošmik M., Delord J.-P., Zasadny X., Geoffrois L., Tao Y., Pointreau Y., Fietkau R., Haderlein M., Mueller A. H., Schroeder U., Wollenberg B., Laban S., Ivanyi P., Gruenwald V., Schafhausen P., Gutfeld O., Gluck I., Popovtzer A., Meirovitz A., Billan S., Brenner B., Popovtzer A., Limon D., Licitra L., Perri F., Caponigro F., Violati M., Ferrari D., Nole F., Bertolini F., Livi L., Ghi M. G., Imarisio I., Tahara M., Homma A., Ueda T., Asada Y., Yamazaki T., Matsumoto K., Fujii T., Ikeda S., Takahashi S., Kinoshita T., Sasaki K., Tsuji A., Ahn M.-J., Cho B. C., Lee K.-W., Lee K. H., Choi M. K., Yun H. J., Hendriks M. P., Oosting S. F., Buter J., Van Meerten E., Graham J., Kawecki A., Debicka I., Maciejczyk A., Pysz M., Filarska D., Hetnał M., Koralewski P., Wygoda A., Składowski K., Talerczyk M., Berrocal Jaime A., Pérez Segura P., Braña García I., Basté Rotllan N., Mesía Nin R., Taberna Sanz M., Iglesias Docampo L., Soria Rivas A., Rueda Domínguez A., Trigo Pérez J. M., Hong R.-L., Li S.-H., Wang H.-M., Yen C.-J., Yang M.-H., Chang Y.-F., Liu Y.-C., Lin J.-C., Ekenel M., Harputluoğlu H., Aksoy S., Özyilkan Ö., Bılıcı A., Şendur M. A. N., Arslan C., Harrington K., Ramkumar S., Gujral D., Stewart S., Powell M., Sibtain A., Roques T., Yip K., Mirza A., Sivaramalingam M., Belman N. D., Agarwala S., Anderson I., Patel A., Maggiore R., Baumgart M., Burtness B., Fidler M. J., Kaur V., Gaughan E., Worden F., Rodriguez C. P., Sukari A., Wong D., Yom S., Walsh W. V., Fiorillo J. A., Yorio J. T., Obara G. S. (2024). Lancet Oncol..

[cit192] Shiravand Y., Khodadadi F., Kashani S. M. A., Hosseini-Fard S. R., Hosseini S., Sadeghirad H., Ladwa R., O’Byrne K., Kulasinghe A. (2022). Curr. Oncol..

[cit193] Robertson A. G., Rendina L. M. (2021). Chem. Soc. Rev..

[cit194] Nanobiotix announces first ever radioenhancer to receive european market approval, https://ml-eu.globenewswire.com/Resource/Download/63f3c5b2-c59b-4e44-abb9-168ef2a5df31, (accessed December 2025)

[cit195] Bonvalot S., Rutkowski P. L., Thariat J., Carrère S., Ducassou A., Sunyach M.-P., Agoston P., Hong A., Mervoyer A., Rastrelli M., Moreno V., Li R. K., Tiangco B., Herraez A. C., Gronchi A., Mangel L., Sy-Ortin T., Hohenberger P., de Baère T., Le Cesne A., Helfre S., Saada-Bouzid E., Borkowska A., Anghel R., Co A., Gebhart M., Kantor G., Montero A., Loong H. H., Vergés R., Lapeire L., Dema S., Kacso G., Austen L., Moureau-Zabotto L., Servois V., Wardelmann E., Terrier P., Lazar A. J., Bovée J. V. M. G., Le Péchoux C., Papai Z. (2019). Lancet Oncol..

[cit196] Le Tourneau C., Hoffmann C., Takacsi-Nagy Z., Liem X., Salas S., Debard A., Finzi L., Farber L. A., Gogishvili M., Kristesashvili G., Makharadze T., Yom S. S. (2022). J. Clin. Oncol..

[cit197] Hu Y., Paris S., Barsoumian H., Abana C. O., He K., Wasley M., Younes A. I., Masrorpour F., Chen D., Yang L., Dunn J. D., Zhang J., Gandhi S., Nguyen Q. N., Cortez M. A., Welsh J. (2021). Int. J. Radiat. Oncol., Biol., Phys..

[cit198] Verry C., Dufort S., Villa J., Gavard M., Iriart C., Grand S., Charles J., Chovelon B., Cracowski J. L., Quesada J. L., Mendoza C., Sancey L., Lehmann A., Jover F., Giraud J. Y., Lux F., Crémillieux Y., McMahon S., Pauwels P. J., Cagney D., Berbeco R., Aizer A., Deutsch E., Loeffler M., Le Duc G., Tillement O., Balosso J. (2021). Radiother. Oncol..

[cit199] NH TherAguix company liquidation on May 2025, https://www.linkedin.com/posts/g%C3%A9raldine-le-duc-801837b1_dear-shareholders-colleagues-collaborators-activity-7333532400267759616-0iq4/, (accessed December 2025)

[cit200] Koshy M., Spiotto M., Feldman L. E., Luke J. J., Fleming G. F., Olson D., Moroney J. W., Nanda R., Rosenberg A., Pearson A. T., Juloori A., Weinberg F., Ray C., Gaba R. C., Chang P. J., Janisch L. A., Xu Z.-Q., Lin W., Weichselbaum R. R., Chmura S. J. (2023). J. Clin. Oncol..

[cit201] Lickliter J. D., Ruben J., Kichenadasse G., Jennens R., Gzell C., Mason R. P., Zhou H., Becker J., Unger E., Stea B. (2023). Cancer Res. Commun..

[cit202] Nimalasena S., Gothard L., Anbalagan S., Allen S., Sinnett V., Mohammed K., Kothari G., Musallam A., Lucy C., Yu S., Nayamundanda G., Kirby A., Ross G., Sawyer E., Castell F., Cleator S., Locke I., Tait D., Westbury C., Wolstenholme V., Box C., Robinson S. P., Yarnold J., Somaiah N. (2020). Int. J. Radiat. Oncol., Biol., Phys..

[cit203] Ogawa Y., Kubota K., Aoyama N., Yamanishi T., Kariya S., Hamada N., Nogami M., Nishioka A., Onogawa M., Miyamura M. (2015). Cancers.

[cit204] Obata S., Ishimaru Y., Miyagi S., Nakatake M., Kuroiwa A., Ohta Y., Kan T., Kanegae S., Inoue Y., Nishizato R., Miyazaki K. (2022). Mol. Clin. Oncol..

[cit205] Li Y., Barmin R. A., Zhang R., Kiessling F., Lammers T., Pallares R. M. (2026). Adv. Drug Delivery Rev..

[cit206] Stupp R., Mason W. P., van den Bent M. J., Weller M., Fisher B., Taphoorn M. J. B., Belanger K., Brandes A. A., Marosi C., Bogdahn U., Curschmann J., Janzer R. C., Ludwin S. K., Gorlia T., Allgeier A., Lacombe D., Cairncross J. G., Eisenhauer E., Mirimanoff R. O. (2005). N. Engl. J. Med..

[cit207] Johannsen M., Thiesen B., Wust P., Jordan A. (2010). Int. J. Hyperthermia.

[cit208] Nanomedicine upscaling for early clinical phases of multimodal cancer therapy, https://cordis.europa.eu/project/id/685795, (accessed December 2025)

[cit209] Vall d'Hebron proposes hyperthermic therapy with magnetic nanoparticles to improve treatment of pancreatic cancer with adenocarcinoma, https://vhir.vallhebron.com/en/society/news/vall-dhebron-proposes-hyperthermic-therapy-magnetic-nanoparticles-improve-treatment-pancreatic-cancer-adenocarcinoma, (accessed December 2025)

[cit210] Vall d’Hebron enrolls the first patient in a clinical trial designed to treat locally advanced pancreatic cancer with nanoparticles, https://www.vallhebron.com/en/news/news/vall-dhebron-enrolls-first-patient-clinical-trial-designed-treat-locally-advanced-pancreatic-cancer-nanoparticles, (accessed December 2025)

[cit211] Díaz-Riascos Z. V., Llaguno-Munive M., Lafuente-Gómez N., Luengo Y., Holmes S., Volatron J., Ibarrola O., Mancilla S., Sarno F., Aguirre J. J., Razafindrakoto S., Southern P., Terán F. J., Keogh A., Salas G., Prina-Mello A., Lacal J. C., del Pozo A., Pankhurst Q. A., Hidalgo M., Gazeau F., Somoza Á., Schwartz S., Abasolo I. (2025). ACS Appl. Mater. Interfaces.

[cit212] Al Sabbagh C., Seguin J., Agapova E., Kramerich D., Boudy V., Mignet N. (2020). Eur. J. Pharm. Biopharm..

[cit213] Regenold M., Bannigan P., Evans J. C., Waspe A., Temple M. J., Allen C. (2022). Nanomedicine.

[cit214] Hossann M., Syunyaeva Z., Schmidt R., Zengerle A., Eibl H., Issels R. D., Lindner L. H. (2012). J. Controlled Release.

[cit215] UroGen Pharma, https://investors.urogen.com/, (accessed December 2025)

[cit216] Solanki R., Bhatia D. (2024). Gels.

[cit217] Prasad S. M., Shishkov D., Mihaylov N. V., Khuskivadze A., Genov P., Terzi V., Kates M., Huang W. C., Louie M. J., Raju S., Burger B., Meads A., Schoenberg M. (2023). J. Urol..

[cit218] Prasad S. M., Huang W. C., Shore N. D., Hu B., Bjurlin M., Brown G., Genov P., Shishkov D., Khuskivadze A., Ganev T., Marchev D., Orlov I., Kopyltsov E., Zubarev V., Nosov A., Komlev D., Burger B., Raju S., Meads A., Schoenberg M. (2025). J. Urol..

[cit219] Zhang L., Yan Y., Gao Y., Chen Y., Yu J., Ren N., Sun L. (2024). Sci. Rep..

[cit220] Lv Y., Cui X., Li T., Liu C., Wang A., Wang T., Zhou X., Li R., Zhang F., Hu Y., Zhang T., Liu Z. (2025). Clin. Exp. Med..

[cit221] Wei Q., Li P., Yang T., Zhu J., Sun L., Zhang Z., Wang L., Tian X., Chen J., Hu C., Xue J., Ma L., Shimura T., Fang J., Ying J., Guo P., Cheng X. (2024). J. Hematol. Oncol..

[cit222] Ma Y., Huang Y., Zhao Y., Zhao S., Xue J., Yang Y., Fang W., Guo Y., Han Y., Yang K., Li Y., Yang J., Fu Z., Chen G., Chen L., Zhou N., Zhou T., Zhang Y., Zhou H., Liu Q., Zhu Y., Zhu H., Xiao S., Zhang L., Zhao H. (2024). Lancet Oncol..

[cit223] Seynhaeve A. L. B., Dicheva B. M., Hoving S., Koning G. A., ten Hagen T. L. M. (2013). J. Controlled Release.

[cit224] Gonzalez-Ochoa E., Veneziani A. C., Oza A. M. (2023). Clin. Med. Insights: Oncol..

[cit225] Matulonis U. A., Lorusso D., Oaknin A., Pignata S., Dean A., Denys H., Colombo N., Van Gorp T., Konner J. A., Marin M. R., Harter P., Murphy C. G., Wang J., Noble E., Esteves B., Method M., Coleman R. L. (2023). J. Clin. Oncol..

[cit226] Moore K. N., Angelergues A., Konecny G. E., Banerjee S. N., Pignata S., Colombo N., Moroney J. W., Cosgrove C., Lee J.-Y., Roszak A., Breuer S., Tromp J. M., Bello-Roufai D., Gilbert L., Miller R., Myers T. K. N., Wang Y., Berkenblit A., Lorusso D., Van Gorp T. (2023). J. Clin. Oncol..

[cit227] US Food and Drug Administration (FDA), https://www.fda.gov/drugs/resources-information-approved-drugs/fda-approves-mirvetuximab-soravtansine-gynx-fra-positive-platinum-resistant-epithelial-ovarian, (accessed December 2025)

[cit228] Johnson M., El-Khoueiry A., Hafez N., Lakhani N., Mamdani H., Rodon J., Sanborn R. E., Garcia-Corbacho J., Boni V., Stroh M., Hannah A. L., Wang S., Castro H., Spira A. (2021). Clin. Cancer Res..

[cit229] Riedel R. F., Chua V., Moradkhani A., Krkyan N., Ahari A., Osada A., Chawla S. P. (2022). Oncologist.

[cit230] Judson I., Verweij J., Gelderblom H., Hartmann J. T., Schöffski P., Blay J.-Y., Kerst J. M., Sufliarsky J., Whelan J., Hohenberger P., Krarup-Hansen A., Alcindor T., Marreaud S., Litière S., Hermans C., Fisher C., Hogendoorn P. C. W., dei Tos A. P., van der Graaf W. T. A. (2014). Lancet Oncol..

[cit231] Tchaparian E., Lin H.-Y., Chen Y., Hunter J. N., Yin S., Ng H., Wu A. (2024). Front. Pharmacol..

[cit232] Tchaparian E., Chu D. (2017). Drug Metab. Pharmacokinet..

[cit233] Pant S., Dragovich T., Lieu C., Jimeno A., Kundranda M., Menter D., Tchaparian E., Chen Y. C., Kopetz S. (2023). Invest. New Drugs.

[cit234] Metselaar J. M., Lammers T. (2020). Drug Delivery Transl. Res..

[cit235] Craig D. J., Nanavaty N. S., Devanaboyina M., Stanbery L., Hamouda D., Edelman G., Dworkin L., Nemunaitis J. J. (2021). Future Oncol..

[cit236] Jørgensen J. T. (2021). Transl. Oncol..

[cit237] Kuznetsov K. M., Cariou K., Gasser G. (2024). Chem. Sci..

[cit238] Fan J., Lennarz R., Zhang K., Mourran A., Meisner J., Xuan M., Göstl R., Herrmann A. (2025). Nat. Commun..

[cit239] Cruz-Nova P., Ancira-Cortez A., Ferro-Flores G., Ocampo-García B., Gibbens-Bandala B. (2022). Pharmaceutics.

[cit240] Chen J., Wang B., Wang Y., Radermacher H., Qi J., Momoh J., Lammers T., Shi Y., Rix A., Kiessling F. (2024). Adv. Sci..

[cit241] Meric-Bernstam F., Larkin J., Tabernero J., Bonini C. (2021). Lancet.

[cit242] Zhang Z., Lu M., Qin Y., Gao W., Tao L., Su W., Zhong J. (2021). Front. Immunol..

[cit243] Novakova A., Morris S. A., Vaiarelli L., Frank S. (2025). Vaccines.

[cit244] Patel D. M. , Patel N. N. and Patel J. K., in Emerging Technologies for Nanoparticle Manufacturing, ed. J. K. Patel and Y. V. Pathak, Springer International Publishing, Cham, 2021, pp. 511–539

[cit245] Domingues C., Santos A., Alvarez-Lorenzo C., Concheiro A., Jarak I., Veiga F., Barbosa I., Dourado M., Figueiras A. (2022). ACS Nano.

[cit246] Dri D. A., Rinaldi F., Carafa M., Marianecci C. (2023). Drug Delivery Transl. Res..

[cit247] Pisanello F., De Vittorio M., Pisano F. (2024). Neurophotonics.

[cit248] Kim K., Min I. S., Kim T. H., Kim D. H., Hwang S., Kang K., Kim K., Park S., Lee J., Cho Y. U., Lee J. W., Yeo W. H., Song Y. M., Jung Y., Yu K. J. (2023). npj Flexible Electron..

[cit249] Demi L., Egan T., Muller M. (2020). Appl. Sci..

[cit250] Vicentini M., Ferrero R., Manzin A. (2024). Int. J. Therm. Sci..

[cit251] Le Tourneau C., Lee J. J., Siu L. L. (2009). J. Natl. Cancer Inst..

[cit252] Antony J. , Design of Experiments for Engineers and Scientists, Elsevier, 2023

[cit253] Rampado R., Peer D. (2023). J. Controlled Release.

[cit254] Rao L., Yuan Y., Shen X., Yu G., Chen X. (2024). Nat. Nanotechnol..

[cit255] Boutet A., Madhavan R., Elias G. J. B., Joel S. E., Gramer R., Ranjan M., Paramanandam V., Xu D., Germann J., Loh A., Kalia S. K., Hodaie M., Li B., Prasad S., Coblentz A., Munhoz R. P., Ashe J., Kucharczyk W., Fasano A., Lozano A. M. (2021). Nat. Commun..

[cit256] Reis M. E., Bettencourt A., Ribeiro H. M. (2022). Front. Med..

[cit257] Bhushan A., Misra P. (2024). Curr. Oncol. Rep..

[cit258] Xue C., Hu S., Gao Z.-H., Wang L., Luo M.-X., Yu X., Li B.-F., Shen Z., Wu Z.-S. (2021). Nat. Commun..

[cit259] Choi H. W., Lim J. H., Kang T., Chung B. G. (2022). Antioxidants.

[cit260] Liang M., Hu S., Han Y., Liu Z., Li C.-P., Hao J., Xue P. (2023). ACS Appl. Mater. Interfaces.

[cit261] Li H., Feng Y., Luo Q., Li Z., Li X., Gan H., Gu Z., Gong Q., Luo K. (2023). Theranostics.

[cit262] Bischof J., Fletcher G., Verkade P., Kuntner C., Fernandez-Rodriguez J., Chaabane L., Rose L. A., Walter A., Vandenbosch M., van Zandvoort M. A. M. J., Zaritsky A., Keppler A., Parsons M. (2024). npj Imaging.

[cit263] Daud M. L., De Simone G. G. (2024). Ecancermedicalscience.

[cit264] Alkahtani M. E., Elbadawi M., Chapman C. A. R., Green R. A., Gaisford S., Orlu M., Basit A. W. (2024). Adv. Healthcare Mater..

[cit265] Di Trani N., Silvestri A., Sizovs A., Wang Y., Erm D. R., Demarchi D., Liu X., Grattoni A. (2020). Lab Chip.

[cit266] Song T., Li K., Wang J., Sun X., Li S., Yang C., Li P. (2025). J. Mater. Chem. B.

[cit267] Yi J., Zou G., Huang J., Ren X., Tian Q., Yu Q., Wang P., Yuan Y., Tang W., Wang C., Liang L., Cao Z., Li Y., Yu M., Jiang Y., Zhang F., Yang X., Li W., Wang X., Luo Y., Loh X. J., Li G., Hu B., Liu Z., Gao H., Chen X. (2023). Nature.

[cit268] Foutz T. J., Wong M. (2022). Biomed. J..

[cit269] Zhou S., Zhao W., Hu J., Mao C., Zhou M. (2023). Adv. Healthcare Mater..

[cit270] Yarali E., Baniasadi M., Zolfagharian A., Chavoshi M., Arefi F., Hossain M., Bastola A., Ansari M., Foyouzat A., Dabbagh A., Ebrahimi M., Mirzaali M. J., Bodaghi M. (2022). Appl. Mater. Today.

[cit271] Zhang K., Zhou Y., Zhang J., Liu Q., Hanenberg C., Mourran A., Wang X., Gao X., Cao Y., Herrmann A., Zheng L. (2024). Nat. Commun..

[cit272] Tang M., Liu Y. H., Xu X. M., Zhang Y. M., Liu Y. (2022). Bioorg. Med. Chem..

[cit273] Abu Serea E. S., Orue I., García J. Á., Lanceros-Méndez S., Reguera J. (2023). ACS Appl. Nano Mater..

[cit274] Gong J., Shi T., Liu J., Pei Z., Liu J., Ren X., Li F., Qiu F. (2023). Biomed. Pharmacother..

[cit275] Kuang G., Ding J., Xie W., Ye Z., Zhang Q. (2025). Int. J. Nanomedicine.

[cit276] Arsuffi B., Siqueira G., Nyström G., Titotto S., Magrini T., Daraio C. (2024). Adv. Funct. Mater..

[cit277] Mazidi Z., Javanmardi S., Naghib S. M., Mohammadpour Z. (2022). Chem. Eng. J..

[cit278] Del Bono F., Di Trani N., Demarchi D., Grattoni A., Motto Ros P. (2025). Lab Chip.

[cit279] Chen Z., Chen J., Jung S., Kim H. Y., Lo Preti M., Laschi C., Ren Z., Sitti M., Full R. J., Yang G. Z. (2025). Matter.

[cit280] Di Trani N., Silvestri A., Bruno G., Geninatti T., Chua C. Y. X., Gilbert A., Rizzo G., Filgueira C. S., Demarchi D., Grattoni A. (2019). Lab Chip.

[cit281] Kim W. S., Khot M. I., Woo H. M., Hong S., Baek D. H., Maisey T., Daniels B., Coletta L. P., Yoon B. J., Jayne D. G., Park S. I. (2022). Nat. Commun..

[cit282] Liang B. J., Pang S., Perttila R., Ma C.-H., Srivastava P., Gaitan B., Sorrin A. J., Fadul N., Rahman I., Ylniemi Z., Roque D. M., Hasan T., Uusimaa P., Huang H.-C. (2023). Sci. Adv..

[cit283] Rama E., Mohapatra S. R., Sugimura Y., Suzuki T., Siebert S., Barmin R., Hermann J., Baier J., Rix A., Lemainque T., Koletnik S., Elshafei A. S., Pallares R. M., Dadfar S. M., Tolba R. H., Schulz V., Jankowski J., Apel C., Akhyari P., Jockenhoevel S., Kiessling F. (2024). Biomaterials.

