# Supplementary material for: Clinical translation and landscape of stimuli-responsive nanomedicines and microscale therapeutics
Source: Chem Soc Rev. 2026 May 29;55(12):6704–38. doi: 10.1039/d6cs00165c (PMC13220073; doi:10.1039/d6cs00165c)
Supplement: CS-055-D6CS00165C-s001 [file CS-055-D6CS00165C-s001.pdf]

Supplementary Information (SI) for Chemical Society Reviews.
This journal is © The Royal Society of Chemistry 2026

Supporting Information

Clinical translation and landscape of stimuli-responsive nanomedicines and microscale therapeutics

Dmytro Kobzev[1]*, Olesia Kulyk[2], Roman Barmin[1], Anatoliy Taratets[2], Roger M. Pallares[1], Fabian Kiessling[1], Twan Lammers[1]*, Quim Peña[1]*

[1] Institute for Experimental Molecular Imaging, Center for Biohybrid Medical Systems, RWTH Aachen University Clinic, Aachen 52074, Germany

[2] Institute of Functional Materials Chemistry of State Scientific Institution "Institute for Single Crystals" of National Academy of Sciences of Ukraine, Kharkiv, 61072, Ukraine

* Corresponding authors: dkobzev@ukaachen.de, tlammers@ukaachen.de, jpena@ukaachen.de

Scheme S1. Keywords search

General setup of literature search.

Accessed: October 2025

Dimensions.ai:

- Search in: Title and abstract;
- Date range: 1980–2024 – total (Fig. 3A); 2014–2024 – last 10 years (Figs. 3B,C)
- Search scheme: therapy type (olive, total number) **AND** material (each specific class)
    - Nanomaterials and microparticles (purple),
    - small molecules (dark blue)

Clinicaltrials.gov:

- Study type: Interventional studies;
- Date range: Study start from 01/01/1980 to 01/01/2025 (Fig. 4A). Study start: from 01/01/2014 to 01/01/2025 (Figs. 4B, 5, 6);
- Search fields: Intervention/treatment (highlighted in olive);
  Other field – for specific material type:
    - Nanomaterials and microparticles (purple),
    - small molecules (dark blue)

1. **Light**

For total search: ("photodynamic therapy" OR "photothermal therapy" OR photoimmunotherapy OR "AuroShell")

For liposomes: AND (liposomes OR (EXPANSION[Term]Visudyne)
For LNP: AND ("Lipid nanoparticle" OR "LNP" OR vesicles* OR "lipid droplets")
For micelles: AND (micelles OR micellar)
For hydrogels: AND (hydrogel OR nanogel)
For polymers: AND polymers NOT micelles NOT liposomes NOT hydrogels
For polysaccharides: AND (chitosan OR saccharides OR carbohydrates OR polysaccharides OR polycarbohydrates OR Dextran OR "Hyaluronic Acid" OR "Pullulan" OR "Cyclodextrins" OR "Alginate" OR Inulin OR Mannans OR dendrimer) NOT hydrogels NOT polymers NOT peptides NOT liposomes NOT micelles
For ADC: AND (Antibody OR "antibody-drug conjugate" OR "ADC" OR "ASP-1929")
For silica/carbon nanoparticles: AND (nanotherapeutics OR nanocarriers OR nanomedicines OR nanoparticles OR nanotubes OR nanorods OR nanomaterials OR nanospheres OR nanocapsules OR nanoshells OR nanovehicles OR nanostructures) AND ("silica" OR "carbon")
For metal nanoparticles: AND (nanotherapeutics OR nanocarriers OR nanomedicines OR nanoparticles OR nanotubes OR nanorods OR nanomaterials OR nanospheres OR nanocapsules OR nanoshells OR nanovehicles OR nanostructures) AND ("Metal" OR "gold" OR "copper" OR "hafnium" OR "ruthenium" OR "iron" OR "metal organic framework" OR MOF OR "gadolinium" OR "silver" OR "zinc" OR "bismuth" OR "titanium" OR "manganese" OR "cobalt" OR "platinum" OR "palladium" OR "AuroShell")
For quantum dots: AND "quantum dots"

For 5-Aminolevulinic acid: AND (5-AVA OR "5-Aminolevulinic acid" OR levulan OR Ameluz OR “Methyl Aminolevulinate” OR “Metvix”)
For porphyrins: AND (porphyrin OR Photofrin OR Verteporfin OR Foscan OR Laserphyrin OR Redaporfin OR Hemoporfin OR Tookad OR padeliporfin OR Fimaporfin OR “chlorin e6” OR bacteriochlorin OR Sinoporphyrin)
AND (phthalocyanine OR ASP-1929 OR IR700 OR RM-1929 OR saratolacan OR Akalux OR Photosens OR PhotoDyn OR Arosurf OR Sicophthal OR Heliostar OR Luzim)
For BODIPY: AND BODIPY
For polymethines: AND (cyanines OR polymethines OR heptamethines OR pentamethines OR squaraines OR “indocyanine green” OR ICG) NOT phthalocyanines

For xanthenes: AND ("Rose Bengal" OR fluorescein OR xanthene OR rhodamine OR “erythrosine b” OR “eosyn”) NOT angiography
For Methylene blue: AND (“Methylene Blue” OR “Toluidine Blue” OR Lumoral)
For ruthenium (II) complexes: AND (TLD-1433 OR Ruvidar OR “Ru(II)” OR ruthenium)

* The *“vesicles”* keyword was excluded from clinicaltrials.org search to exclude false-positive results.

2. Ultrasound

For total search: ("ultrasound-responsive" OR "ultrasound-triggered" OR "ultrasound-mediated" OR "sonodynamic therapy" OR ("focused ultrasound" AND microbubbles) OR SonoVue OR Sonazoid OR Lumason OR Definity) AND (therapy OR treatment OR thermotherapy OR ablation OR sonopermeation OR sonotherapy) NOT (“contrast enhanced ultrasound” OR “CEUS” OR surgery OR acoustic OR echocardiography)

For liposomes: AND liposomes
For LNP: AND (“Lipid nanoparticle” OR “LNP” OR vesicles)
For micelles: AND (micelles OR micellar)
For hydrogels: AND (Hydrogel OR Nanogel)
For polymers: AND polymers NOT micelles NOT liposomes NOT hydrogels NOT microbubbles NOT nanobubbles
For polysaccharides: AND (chitosan OR saccharides OR carbohydrates OR polysaccharides OR polycarbohydrates OR Dextran OR “Hyaluronic Acid” OR “Pullulan” OR “Cyclodextrins” OR “Alginate” OR Inulin OR Mannans) NOT hydrogels NOT polymers NOT peptides NOT liposomes NOT micelles
For ADC: AND (Antibody OR “antibody-drug conjugate” OR “ADC”)
For silica/carbon nanoparticles: AND (nanotherapeutics OR nanocarriers OR nanomedicines OR nanoparticles OR nanotubes OR nanorods OR nanomaterials OR nanospheres OR nanocapsules OR nanoshells OR nanovehicles OR nanostructures) AND (“silica” OR “carbon”)
For metal nanoparticles: AND (nanotherapeutics OR nanocarriers OR nanomedicines OR nanoparticles OR nanotubes OR nanorods OR nanomaterials OR nanospheres OR nanocapsules OR nanoshells OR nanovehicles OR nanostructures) AND (“Metal” OR “gold” OR “copper” OR “hafnium” OR “ruthenium” OR “iron” OR “metal organic framework” OR MOF OR “gadolinium” OR “silver” OR “zinc” OR “bismuth” OR “titanium” OR “manganese” OR “cobalt” OR “platinum” OR “palladium”)

For microbubbles: AND (microbubbles OR SonoVue OR Sonazoid OR Lumason OR Definity OR Optison)
For nanobubbles: AND nanobubbles
For nanodroplets: AND nanodroplets

For fluorescent dyes: AND (5-AVA OR "5-Aminolevulinic acid" OR levulan OR Ameluz OR “Methyl Aminolevulinate” OR “Metvix” OR SONALA OR porphyrin OR Photofrin OR Visudyne OR Foscan OR Laserphyrin OR Redaporfin OR Hemoporfin OR Tookad OR padeliporfin OR Fimaporfin OR “chlorin e6” OR bacteriochlorin OR Sinoporphyrin OR phthalocyanine OR ASP-1929 OR IR700 OR RM-1929 OR saratolacan OR Akalux OR Photosens OR PhotoDyn OR Arosurf OR Sicophthal OR Heliostar OR Luzim OR BODIPY OR cyanines OR polymethines OR heptamethines OR pentamethines OR squaraines OR “indocyanine green” OR ICG OR "Rose Bengal" OR fluorescein OR xanthene OR rhodamine OR “erythrosine b” OR “Methylene Blue” OR “Toluidine Blue” OR Lumoral OR TLD-1433 OR Ruvidar OR “Ru(II)”)

3. Magnetic field

For total search: ("magnetic-responsive" OR "magnetically-responsive" OR "magneto-responsive" OR "magnetic field-triggered" OR "magnetic field-activated" OR "magnetically-sensitive" OR "magneto-thermal" OR "magnetic-induced" OR "magnetic hyperthermia" OR “magnetic nanoparticle hyperthermia” OR “Nanotherm” OR “MagForce” OR “spinning magnetic field”) AND (therapy OR treatment OR thermotherapy OR ablation OR magnetotherapy)

For liposomes: AND liposomes
For LNP: AND (“Lipid nanoparticle” OR “LNP” OR vesicles)
For micelles: AND (micelles OR micellar)
For hydrogels: AND (Hydrogel OR Nanogel)
For polymers: AND polymers NOT micelles NOT liposomes NOT hydrogels
For polysaccharides: AND (chitosan OR saccharides OR carbohydrates OR polysaccharides OR polycarbohydrates OR Dextran OR “Hyaluronic Acid” OR “Pullulan” OR “Cyclodextrins” OR “Alginate” OR Inulin OR Mannans) NOT hydrogels NOT polymers NOT peptides NOT liposomes NOT micelles
For ADC: AND (Antibody OR “antibody-drug conjugate” OR “ADC”)
For silica/carbon nanoparticles: AND (nanotherapeutics OR nanocarriers OR nanomedicines OR nanoparticles OR nanotubes OR nanorods OR nanomaterials OR nanospheres OR nanocapsules OR nanoshells OR nanovehicles OR nanostructures) AND (“silica” OR “carbon”)
For iron oxide nanoparticles: AND (nanotherapeutics OR nanocarriers OR nanomedicines OR nanoparticles OR nanotubes OR nanorods OR nanomaterials OR nanospheres OR nanocapsules OR nanoshells OR nanovehicles OR nanostructures) AND (“iron” OR "iron oxide" OR "Fe3O4" OR magnetite OR "Fe2O3" OR “superparamagnetic iron oxide” OR “SPION”)
For other metal nanoparticles: AND (nanotherapeutics OR nanocarriers OR nanomedicines OR nanoparticles OR nanotubes OR nanorods OR nanomaterials OR nanospheres OR nanocapsules OR nanoshells OR nanovehicles OR nanostructures) AND (“Metal” OR “gold” OR “copper” OR “hafnium” OR “ruthenium” OR “metal organic framework” OR MOF OR “gadolinium” OR “silver” OR “zinc” OR “bismuth” OR “titanium” OR “manganese” OR “cobalt” OR “platinum” OR “palladium”) NOT

"iron oxide" NOT "Fe3O4" NOT magnetite NOT "Fe2O3" NOT “superparamagnetic iron oxide” NOT “SPION”

4. Radiation

For total search: (“radiation-responsive” OR “radiation-sensitive” OR “radiation-enhanced” OR “radiation-triggered” OR radiosensitizers OR radioenhancers OR “activated by radiation” OR “AGuIX”) AND (radiotherapy OR “radiation therapy”) NOT (radioligand OR “177Lu-PSMA-617” OR radiotracer OR “radiation protective” OR Photobiomodulation OR “Photodynamic therapy” OR "artificial intelligence" OR tooth OR Behavioral OR postoperative OR nutrition OR “F18” OR “Diagnostic Test” OR “ERCP” OR “acupuncture” OR "Radiofrequency" OR "TYRO" OR "Vitamin D" OR “Rose Bengal”) NOT (EXPANSION[Term]("post-radiation" OR "postsurgical" OR "surgery"* OR "predict"* OR "Post-Prostatectomy" OR "Post-stroke" OR “imaging quality” OR “breastfeeding” OR “images” OR “dietary supplement”))

For liposomes: AND liposomes
For LNP: AND (“Lipid nanoparticle” OR “LNP” OR vesicles)
For micelles: AND (micelles OR micellar)
For hydrogels: AND (Hydrogel OR Nanogel)
For polymers: AND polymers NOT micelles NOT liposomes NOT hydrogels
For polysaccharides: AND (chitosan OR saccharides OR carbohydrates OR polysaccharides OR polycarbohydrates OR Dextran OR “Hyaluronic Acid” OR “Pullulan” OR “Cyclodextrins” OR “Alginate” OR Inulin OR Mannans) NOT hydrogels NOT polymers NOT peptides NOT liposomes NOT micelles
For ADC: AND (Antibody OR “antibody-drug conjugate” OR “ADC”)
For silica/carbon nanoparticles: AND (nanotherapeutics OR nanocarriers OR nanomedicines OR nanoparticles OR nanotubes OR nanorods OR nanomaterials OR nanospheres OR nanocapsules OR nanoshells OR nanovehicles OR nanostructures) AND (“silica” OR “carbon”)
For metal nanoparticles: AND (nanotherapeutics OR nanocarriers OR nanomedicines OR nanoparticles OR nanotubes OR nanorods OR nanomaterials OR nanospheres OR nanocapsules OR nanoshells OR nanovehicles OR nanostructures) AND (Hensify OR NBTXR3 OR “AGuIX” OR “Metal” OR “gold” OR “copper” OR “hafnium” OR “ruthenium” OR “iron” OR “metal organic framework” OR MOF OR “gadolinium” OR “silver” OR “zinc” OR “bismuth” OR “titanium” OR “manganese” OR “cobalt” OR “platinum” OR “palladium”)

For chemotherapeutic drugs: AND (chemotherapy OR "targeted drug delivery" OR "drug delivery" OR "drug release" OR “cytotoxic agent” OR “payload” OR prodrug OR “therapeutic agent” OR Paclitaxel OR Cisplatin OR Carboplatin OR Fluorouracil OR Capecitabine OR Gemcitabine OR doxorubicin) NOT (Hensify OR NBTXR3 OR “AGuIX” OR “Metal” OR “gold” OR “copper” OR “hafnium” OR “ruthenium” OR “iron” OR “metal organic framework” OR “MOF” OR “gadolinium” OR “silver” OR “zinc” OR “bismuth” OR KORTUC OR “AGuIX”)
For other organic molecules: AND (“Ropidoxuridine” OR “Pirfenidone” OR “Tranilast”)
For hydrogen peroxide: AND (“hydrogen peroxide” OR “Kochi oxydol” OR KORTUC)

* The *NOT (”surgery” OR “predict”)* keywords were included only for clinicaltrials.org search to exclude false-positive results.

5. Heat

For total search: (thermosensitive OR heat-sensitive) AND (therapy OR treatment OR hyperthermia OR chemotherapy OR thermotherapy) AND (drug OR payload OR prodrug OR “therapeutic agent” OR “cytotoxic agent”) NOT “pain”*

For liposomes: AND (liposomes OR ThermoDox)
For LNP: AND (“Lipid nanoparticle” OR “LNP” OR vesicles)
For micelles: AND (micelles OR micellar)
For hydrogels: AND (Hydrogel OR Nanogel)
For polymers: AND polymers NOT micelles NOT liposomes NOT hydrogels
For polysaccharides: AND (chitosan OR saccharides OR carbohydrates OR polysaccharides OR polycarbohydrates OR Dextran OR “Hyaluronic Acid” OR “Pullulan” OR “Cyclodextrins” OR “Alginate” OR Inulin OR Mannans) NOT hydrogels NOT polymers NOT peptides NOT liposomes NOT micelles
For ADC: AND (Antibody OR “antibody-drug conjugate” OR “ADC”)
For silica/carbon nanoparticles: AND (nanotherapeutics OR nanocarriers OR nanomedicines OR nanoparticles OR nanotubes OR nanorods OR nanomaterials OR nanospheres OR nanocapsules OR nanoshells OR nanovehicles OR nanostructures) AND (“silica” OR “carbon”) NOT (NIR OR "photothermal" OR "magnetic" OR "near infrared" OR ultrasound OR light)
For metal nanoparticles: AND (nanotherapeutics OR nanocarriers OR nanomedicines OR nanoparticles OR nanotubes OR nanorods OR nanomaterials OR nanospheres OR nanocapsules OR nanoshells OR nanovehicles OR nanostructures) AND (“Metal” OR “gold” OR “copper” OR “hafnium” OR “ruthenium” OR “iron” OR “metal organic framework” OR MOF OR “gadolinium” OR “silver” OR “zinc” OR “bismuth” OR “titanium” OR “manganese” OR “cobalt” OR “platinum” OR “palladium”) NOT (NIR OR "photothermal" OR "magnetic" OR "near infrared" OR ultrasound OR light)

* The *NOT “pain”* keyword was included only for clinicaltrials.org search to exclude false-positive results**.**

6. Internal stimuli

For total search: ("targeted drug delivery" OR "drug delivery" OR "drug release" OR “cytotoxic agent” OR “cytotoxic drug” OR “payload” OR prodrug OR “therapeutic agent” OR "chemotherapy" OR "cancer therapy" OR “cancer treatment” OR “responsive drug” OR "encapsulated drug" OR “carrier” OR “antibody-drug conjugates” OR “NC-6300”) AND (“internal stimuli” OR “pH responsive” OR “pH sensitive” OR “pH gradient” OR “pH triggered” OR (“Enzyme responsive” OR “Enzyme hydrolysis” OR “Enzymatic cleavage” OR “Enzyme triggered” OR cathepsin OR caspase OR esterase OR "matrix metalloproteinase" OR hyaluronidase NOT (inhibitor OR inhibition)) OR “reduction-oxidation responsive” OR “RedOx responsive” OR glutathione OR GSH OR “RedOx triggered” OR “disulfide reduction” OR “thiol sensitive” OR “oxidative stress-responsive” OR “antibody-drug conjugates” OR “NC-6300”) NOT (Behavioral OR “Diagnostic Test” OR diagnostics OR environmental OR vaccine)

For liposomes: AND liposomes
For LNP: AND ("Lipid nanoparticles" OR "LNP" OR vesicles OR "lipid droplets")
For micelles: AND (micelles OR micellar)
For hydrogels: AND (Hydrogel OR Nanogel)
For polymers: AND polymers NOT micelles NOT liposomes NOT hydrogels
For polysaccharides: AND (chitosan OR saccharides OR carbohydrates OR polysaccharides OR polycarbohydrates OR Dextran OR "Pullulan" OR "Cyclodextrins" OR "Alginate" OR Inulin OR Mannans) NOT hydrogels NOT polymers NOT peptides NOT liposomes NOT micelles
For ADC: AND (antibody OR "antibody-drug conjugate" OR "ADC")
For silica/carbon nanoparticles: AND (nanotherapeutics OR nanocarriers OR nanomedicines OR nanoparticles OR nanotubes OR nanorods OR nanomaterials OR nanospheres OR nanocapsules OR nanoshells OR nanovehicles OR nanostructures) AND ("silica" OR "carbon")
For metal nanoparticles: AND (nanotherapeutics OR nanocarriers OR nanomedicines OR nanoparticles OR nanotubes OR nanorods OR nanomaterials OR nanospheres OR nanocapsules OR nanoshells OR nanovehicles OR nanostructures) AND ("Metal" OR "gold" OR "copper" OR "hafnium" OR "ruthenium" OR "iron" OR "metal organic framework" OR MOF OR "gadolinium" OR "silver" OR "zinc" OR "bismuth" OR "titanium" OR "manganese" OR "cobalt" OR "platinum" OR "palladium")
